# Supplementary material for: The modeled distribution of corals and sponges surrounding the Salas y Gómez and Nazca ridges with implications for high seas conservation
Source: PeerJ. 2021 Sep 24;9:e11972. doi: 10.7717/peerj.11972 (PMC8475544; doi:10.7717/peerj.11972)
Supplement: Supplemental Information 1 — Depth was excluded from the models but is included here for reference. Correlations greater than 0.6 are highlighted in yellow, greater than 0.7 in orange, and greater than 0.8 in red. [file peerj-09-11972-s001.docx]

|  | Ω_A_ | Depth | Diss. Oxygen | Nitrate | Phosphate | POC | Silicate | Slope | TPI-40000 | VRM-21 |
| --- | --- | --- | --- | --- | --- | --- | --- | --- | --- | --- |
| Ω_A_ | - | 0.851 | -0.509 | -0.373 | 0.145 | 0.769 | -0.784 | 0.004 | 0.307 | 0.176 |
| Depth | 0.851 | - | -0.370 | -0.190 | 0.195 | 0.417 | -0.625 | 0.012 | 0.449 | 0.243 |
| Diss. Oxygen | -0.509 | -0.370 | - | -0.308 | -0.559 | -0.560 | 0.156 | -0.003 | -0.213 | -0.018 |
| Nitrate | -0.373 | -0.190 | -0.308 | - | 0.451 | -0.364 | 0.538 | 0.002 | 0.021 | -0.058 |
| Phosphate | 0.145 | 0.195 | -0.559 | 0.451 | - | 0.083 | 0.089 | 0.016 | 0.177 | 0.007 |
| POC | 0.769 | 0.417 | -0.560 | -0.364 | 0.083 | - | -0.684 | 0.004 | 0.052 | -0.006 |
| Silicate | -0.784 | -0.625 | 0.156 | 0.538 | 0.089 | -0.684 | - | -0.028 | -0.200 | -0.109 |
| Slope | 0.004 | 0.012 | -0.003 | 0.002 | 0.016 | 0.004 | -0.028 | - | -0.006 | 0.021 |
| TPI-40000 | 0.307 | 0.449 | -0.213 | 0.021 | 0.177 | 0.052 | -0.200 | -0.006 | - | 0.203 |
| VRM-21 | 0.176 | 0.243 | -0.018 | -0.058 | 0.007 | -0.006 | -0.109 | 0.021 | 0.203 | - |
