# Supplementary material for: The modeled distribution of corals and sponges surrounding the Salas y Gómez and Nazca ridges with implications for high seas conservation
Source: PeerJ. 2021 Sep 24;9:e11972. doi: 10.7717/peerj.11972 (PMC8475544; doi:10.7717/peerj.11972)
Supplement: Supplemental Information 2 — The percentage of highly suitable area in each feature class is given. Note that geomorphological features overlap, so percentages may add to more than 100%. See Fig. S21 for a map of geomorphological features. Only dominant features in the study were considered. Geomorphological classifications are from Harris et al. (2014). [file peerj-09-11972-s002.docx]

| Feature | Demosponge | Glass Sponge | Stony Coral |
| --- | --- | --- | --- |
| Canyon | 0.0 | 0.0 | 0.0 |
| Escarpment | 68.1 | 99.9 | 75.1 |
| Guyot | 14.7 | 1.0 | 18.7 |
| Plateau | 9.9 | 0.0 | 10.0 |
| Ridge | 72.8 | 78.6 | 75.8 |
| Seamount | 59.9 | 76.5 | 59.7 |
| Spreading Ridge | 0.0 | 1.0 | 0.5 |
| Trench | 0.0 | 0.0 | 0.0 |
