# Supplementary material for: The modeled distribution of corals and sponges surrounding the Salas y Gómez and Nazca ridges with implications for high seas conservation
Source: PeerJ. 2021 Sep 24;9:e11972. doi: 10.7717/peerj.11972 (PMC8475544; doi:10.7717/peerj.11972)
Supplement: Supplemental Information 3 [file peerj-09-11972-s003.docx]

| Family | No. Genera | No. Records |
| --- | --- | --- |
| Caryophylliidae | 10 | 91 |
| Deltocyathidae | 1 | 18 |
| Dendrophylliidae | 3 | 6 |
| Flabellidae | 3 | 10 |
| Fungiacyathidae | 1 | 10 |
| Oculinidae | 1 | 16 |
| Pocilloporidae | 1 | 4 |
| Poritidae | 1 | 4 |
| Rhizangiidae | 2 | 9 |
| Stenocyathidae | 1 | 2 |
| Turbinoliidae | 2 | 6 |
| Other | - | 57 |
| Total | 26 | 233 |
