# Supplementary material for: The modeled distribution of corals and sponges surrounding the Salas y Gómez and Nazca ridges with implications for high seas conservation
Source: PeerJ. 2021 Sep 24;9:e11972. doi: 10.7717/peerj.11972 (PMC8475544; doi:10.7717/peerj.11972)
Supplement: Supplemental Information 4 [file peerj-09-11972-s004.docx]

| Order | No. Families | No. Genera | No. Records |
| --- | --- | --- | --- |
| Axinellida | 2 | 3 | 8 |
| Dendroceratida | 2 | 2 | 9 |
| Dictyoceratida | 3 | 4 | 21 |
| Halichondrida | 1 | 1 | 2 |
| Haplosclerida | 1 | 1 | 7 |
| Poecilosclerida | 5 | 8 | 50 |
| Polymastiida | 1 | 2 | 6 |
| Suberitida | 2 | 4 | 24 |
| Tetractinellida | 1 | 1 | 1 |
| Other | - | - | 147 |
| Total | 18 | 26 | 275 |
