# Supplementary material for: The modeled distribution of corals and sponges surrounding the Salas y Gómez and Nazca ridges with implications for high seas conservation
Source: PeerJ. 2021 Sep 24;9:e11972. doi: 10.7717/peerj.11972 (PMC8475544; doi:10.7717/peerj.11972)
Supplement: Supplemental Information 5 [file peerj-09-11972-s005.docx]

| Order | No. Families | No. Genera | No. Records |
| --- | --- | --- | --- |
| Amphidiscosida | 2 | 3 | 36 |
| Hexactinosida | 1 | 1 | 1 |
| Lyssacinosida | 2 | 6 | 24 |
| Sceptrulophora | 1 | 1 | 6 |
| Other | - | - | 67 |
| Total | 6 | 11 | 134 |
