# Supplementary figures and images for: The modeled distribution of corals and sponges surrounding the Salas y Gómez and Nazca ridges with implications for high seas conservation
Source: PeerJ. 2021 Sep 24;9:e11972. doi: 10.7717/peerj.11972 (PMC8475544; doi:10.7717/peerj.11972)

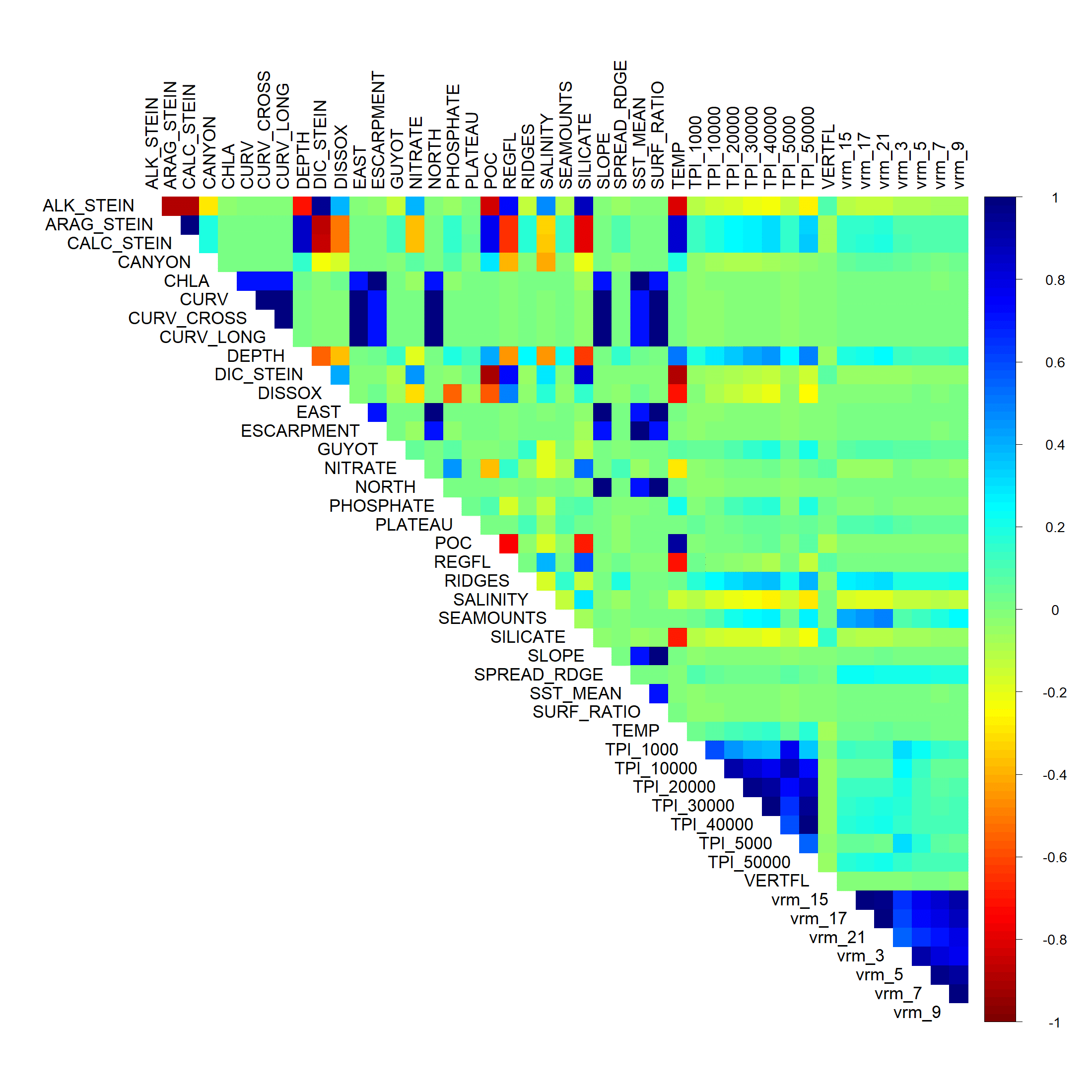

Supplement: Supplemental Information 6 — See Main Text Table 1 for variable descriptions. [file peerj-09-11972-s006.png]

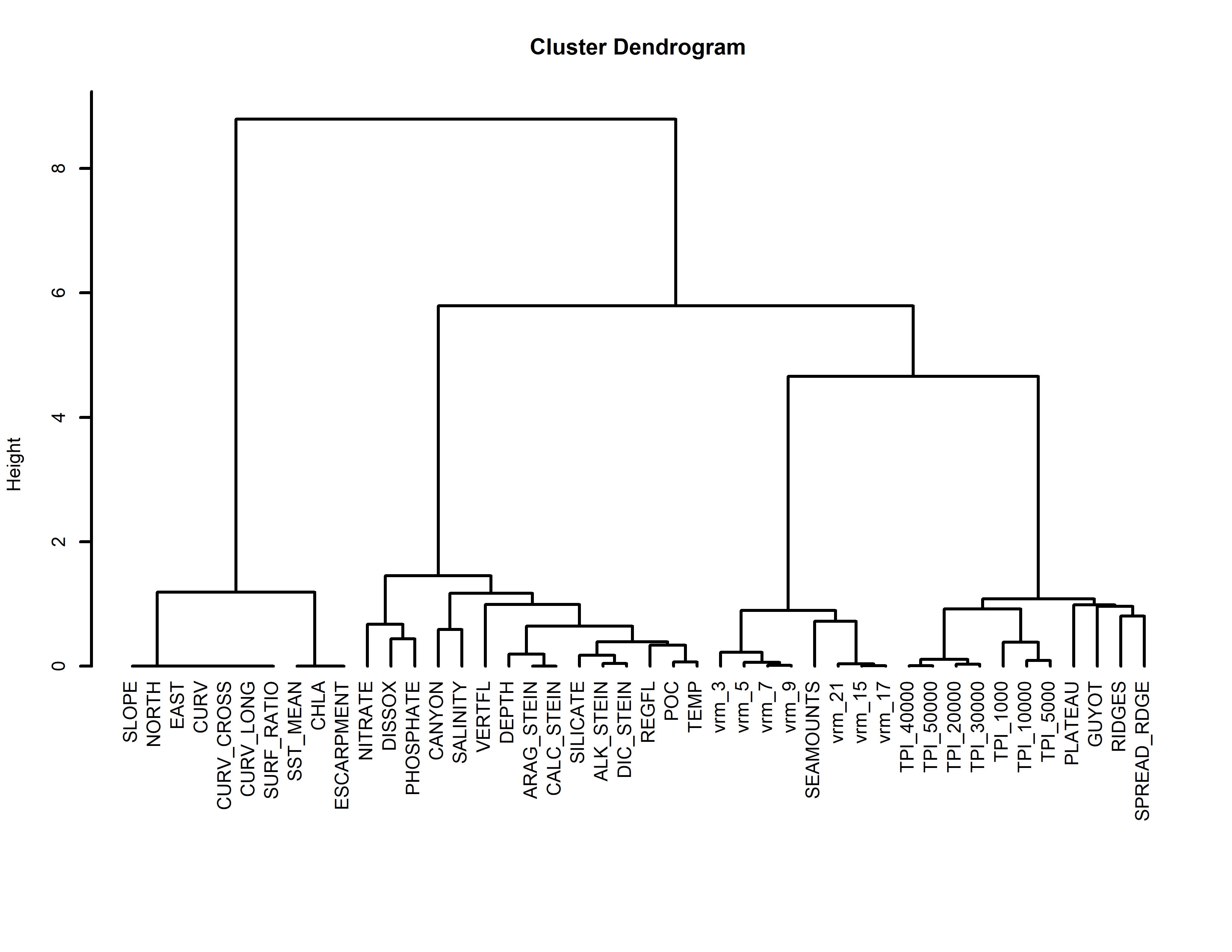

Supplement: Supplemental Information 7 — Variables containing similar information cluster closer together. See Main Text Table 1 for variable descriptions. [file peerj-09-11972-s007.png]

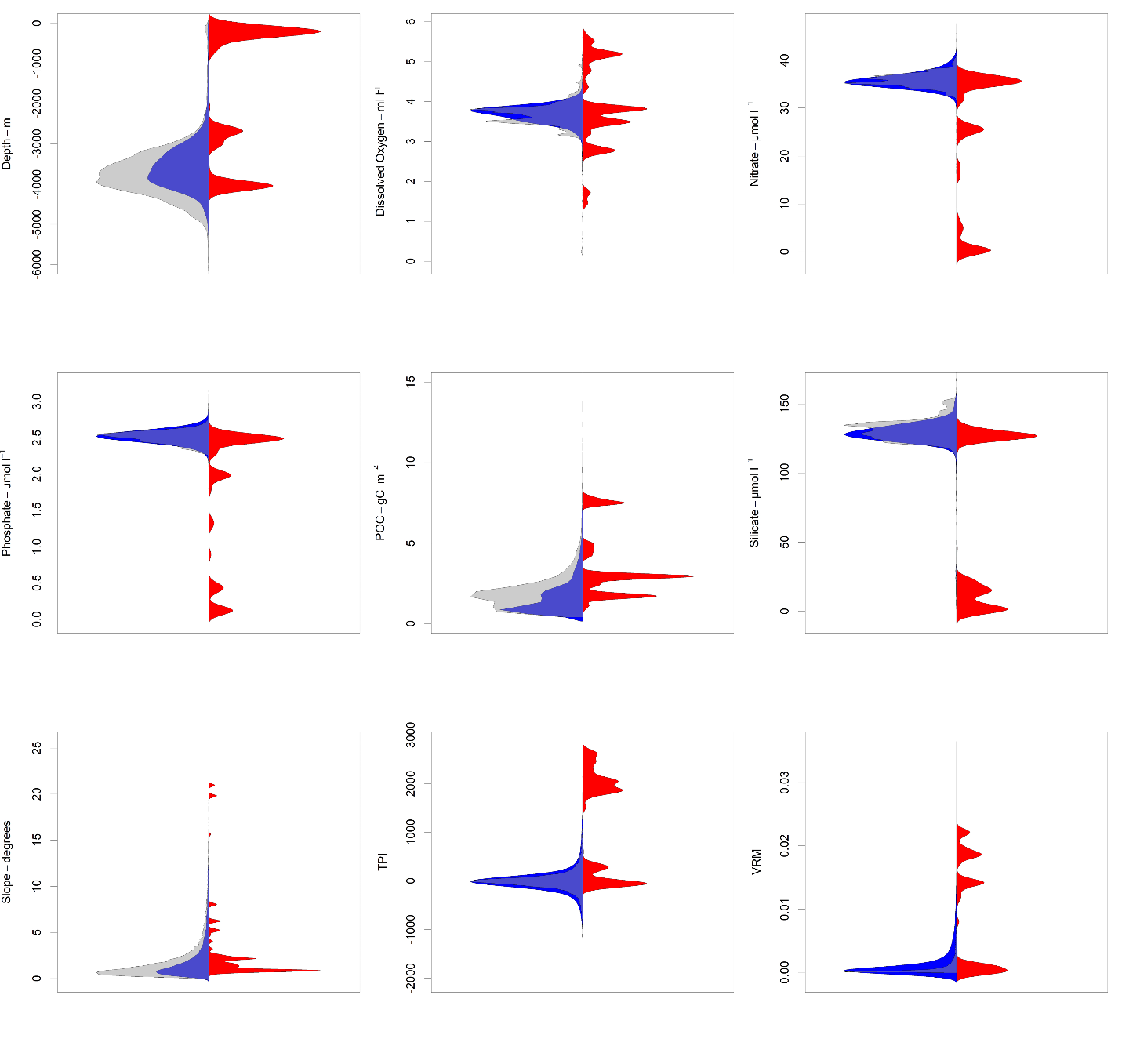

Supplement: Supplemental Information 8 — Occurrences are shown in red, the sample-bias corrected set of pseudoabsences (n=10,000) are shown in blue, and a randomly selected set of points (n=10,000) are shown in grey. [file peerj-09-11972-s008.png]

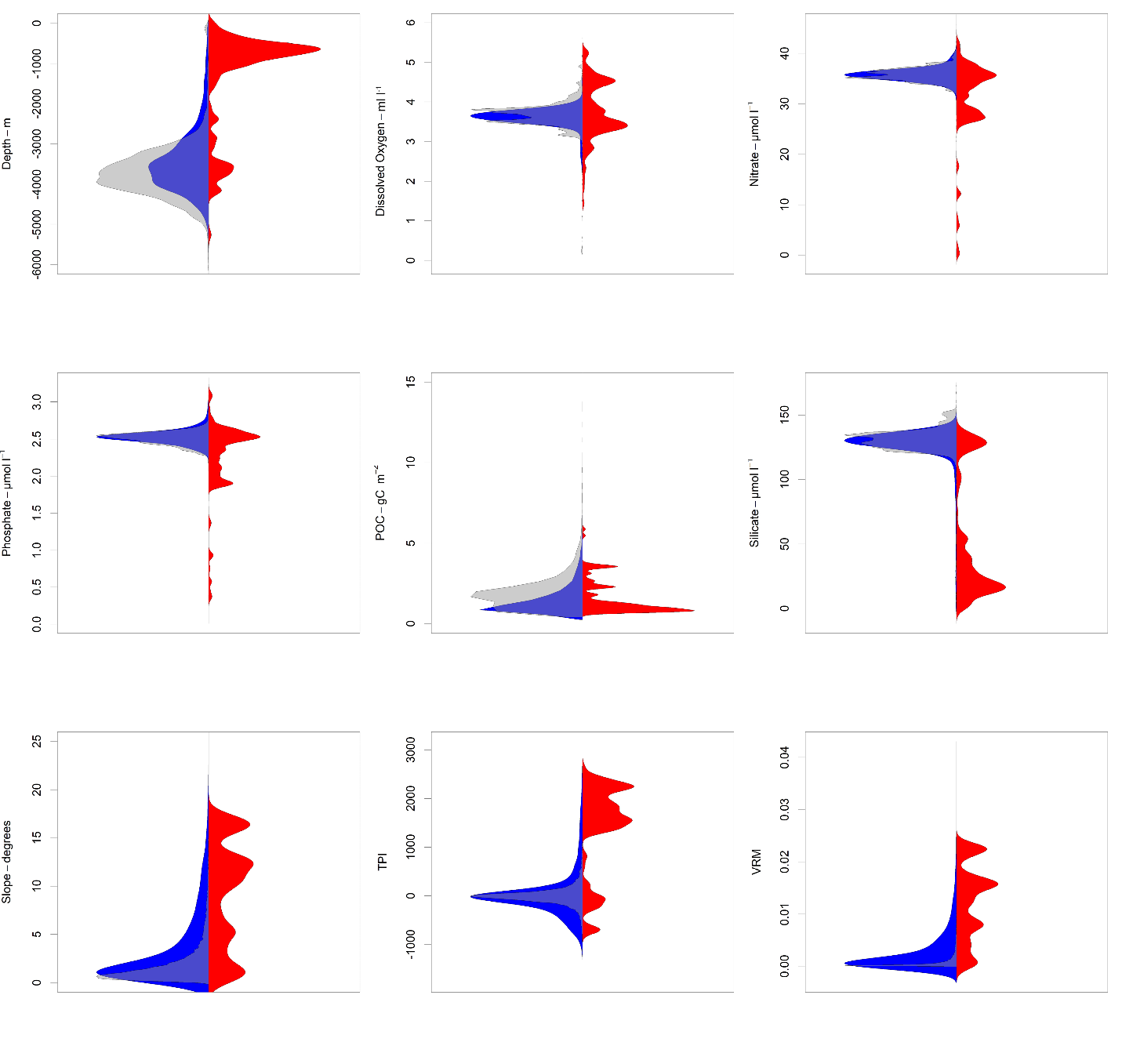

Supplement: Supplemental Information 9 — Occurrences are shown in red, the sample-bias corrected set of pseudoabsences (n=10,000) are shown in blue, and a randomly selected set of points (n=10,000) are shown in grey. [file peerj-09-11972-s009.png]

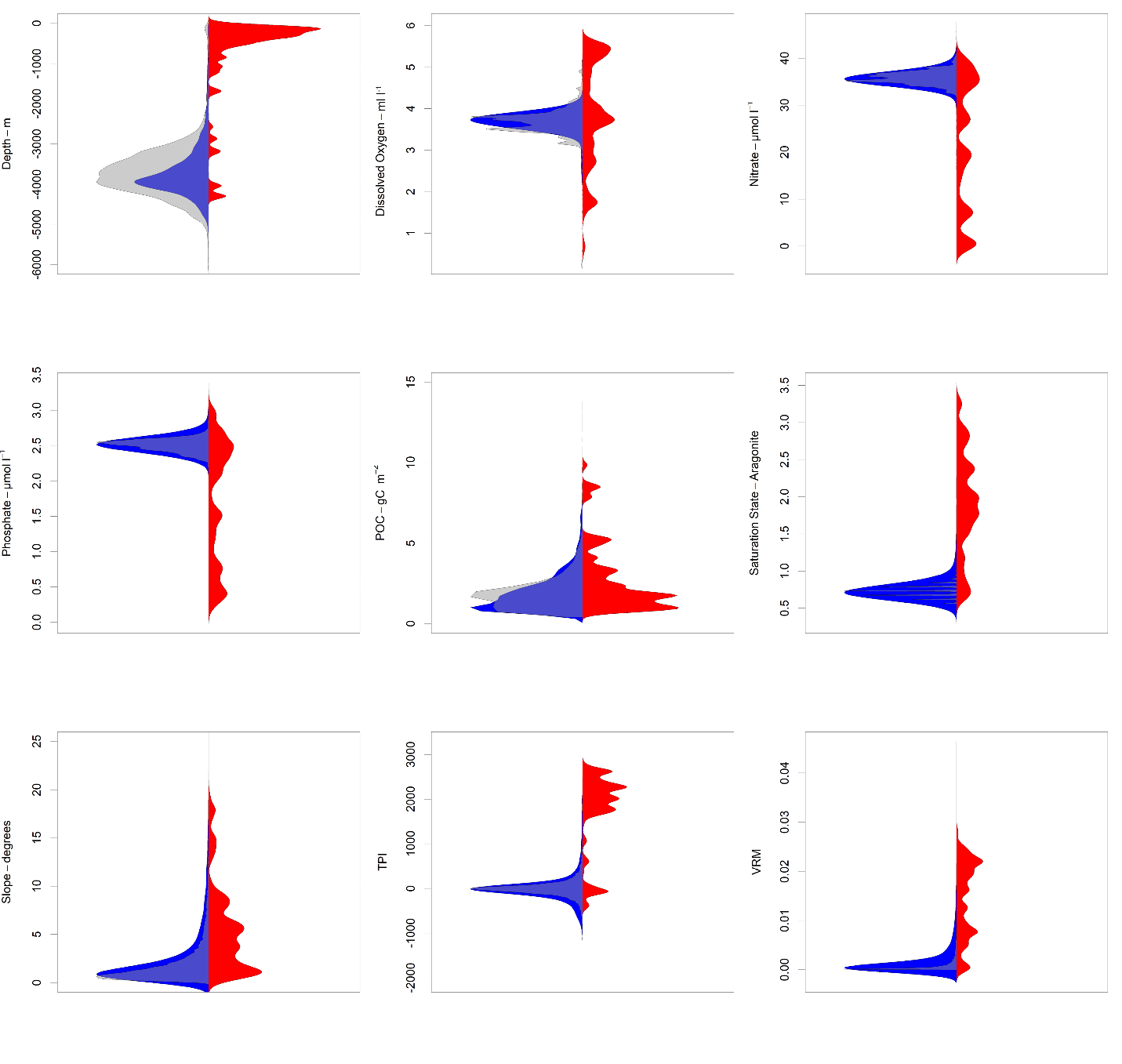

Supplement: Supplemental Information 10 — Occurrences are shown in red, the sample-bias corrected set of pseudoabsences (n=10,000) are shown in blue, and a randomly selected set of points (n=10,000) are shown in grey. [file peerj-09-11972-s010.png]

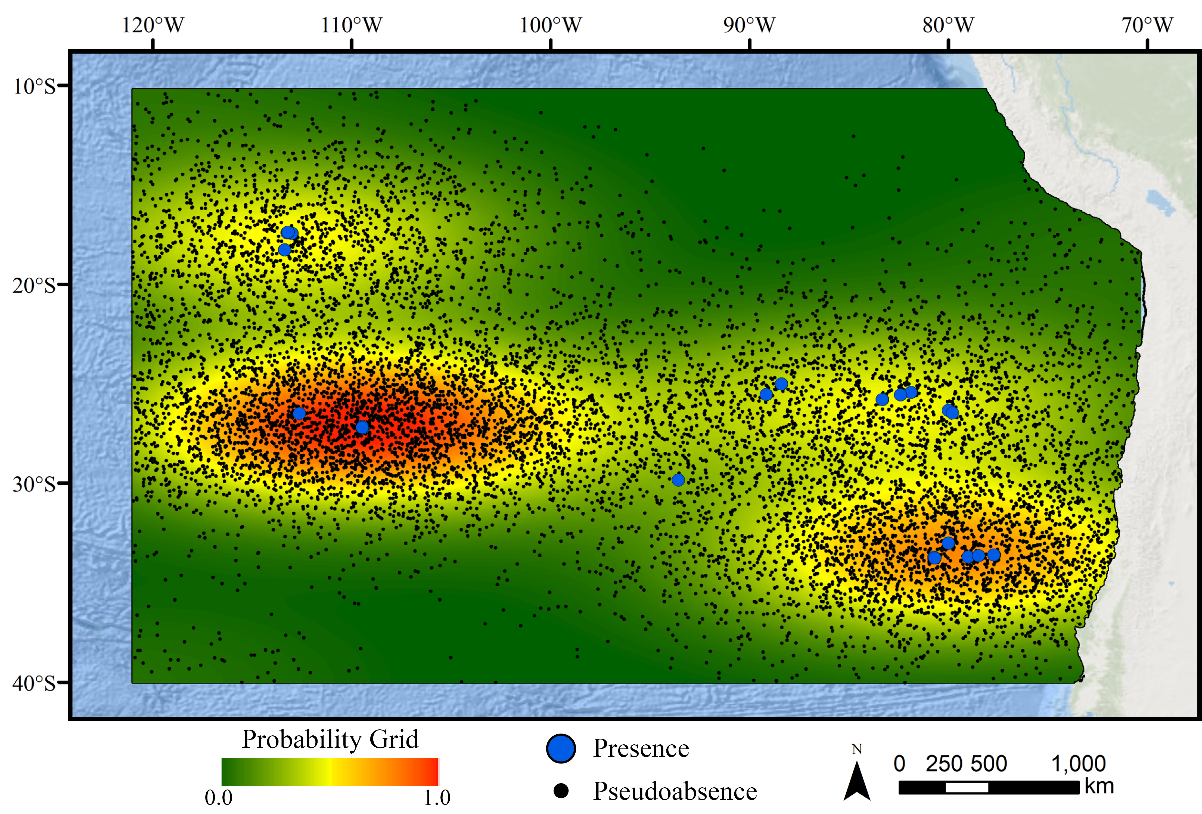

Supplement: Supplemental Information 11 — Pseudoabsences (n=10,000) were selected using the density estimate as a probability with the goal of reducing the effects of sampling bias on model performance. [file peerj-09-11972-s011.png]

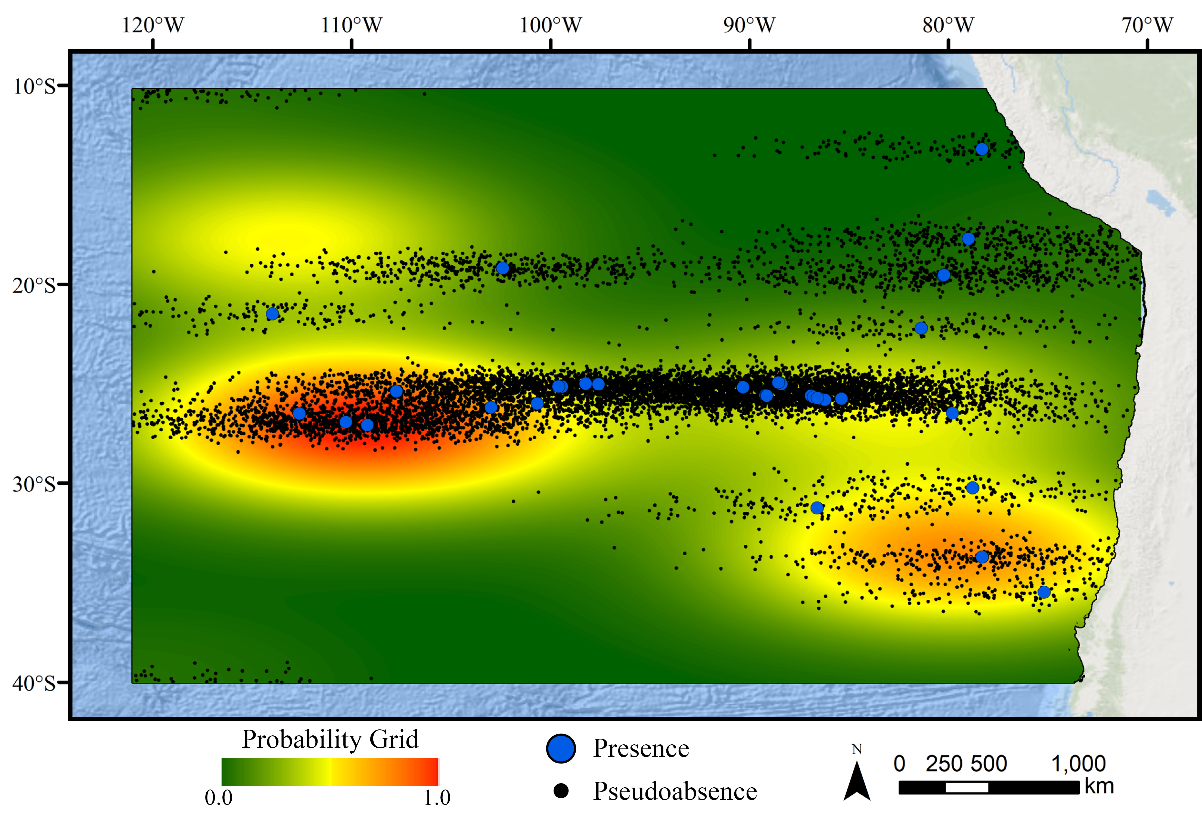

Supplement: Supplemental Information 12 — Pseudoabsences (n=10,000) were selected using the density estimate as a probability with the goal of reducing the effects of sampling bias on model performance. [file peerj-09-11972-s012.png]

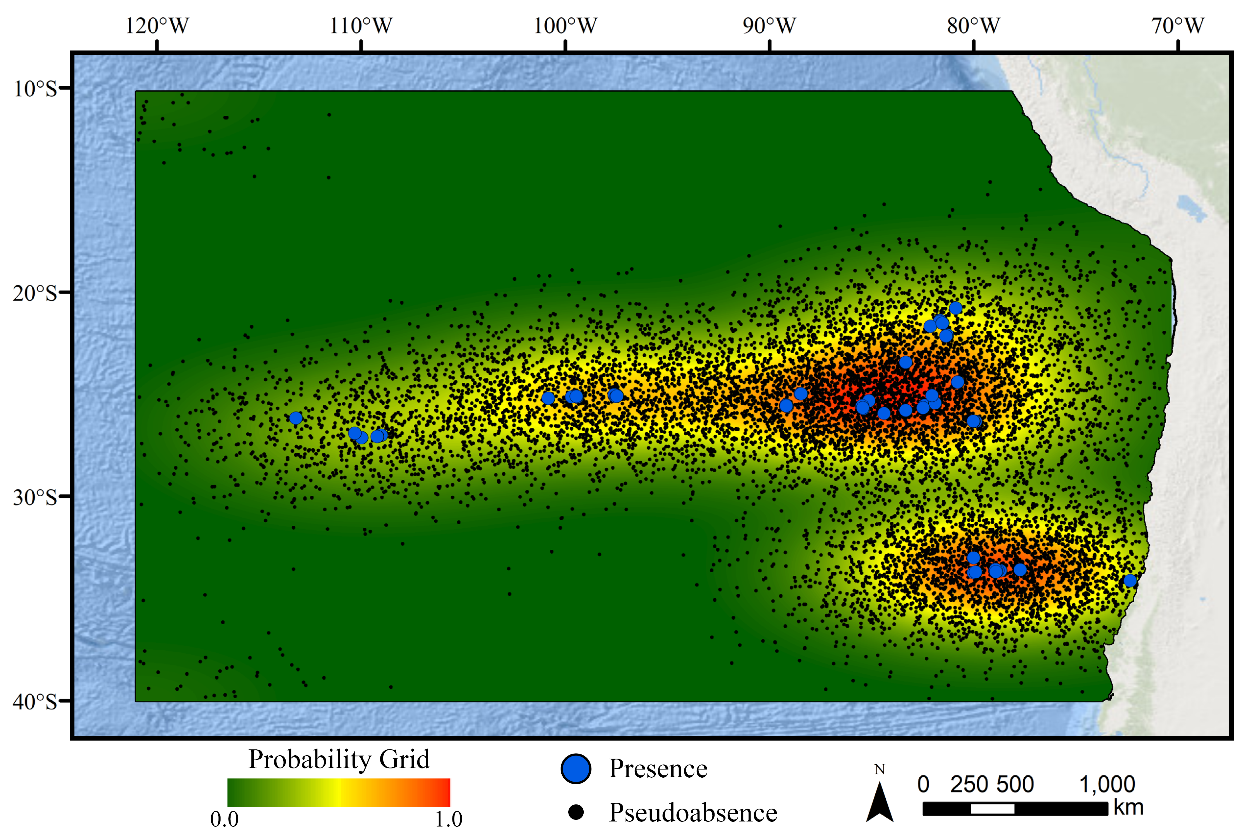

Supplement: Supplemental Information 13 — Pseudoabsences (n=10,000) were selected using the density estimate as a probability with the goal of reducing the effects of sampling bias on model performance. [file peerj-09-11972-s013.png]

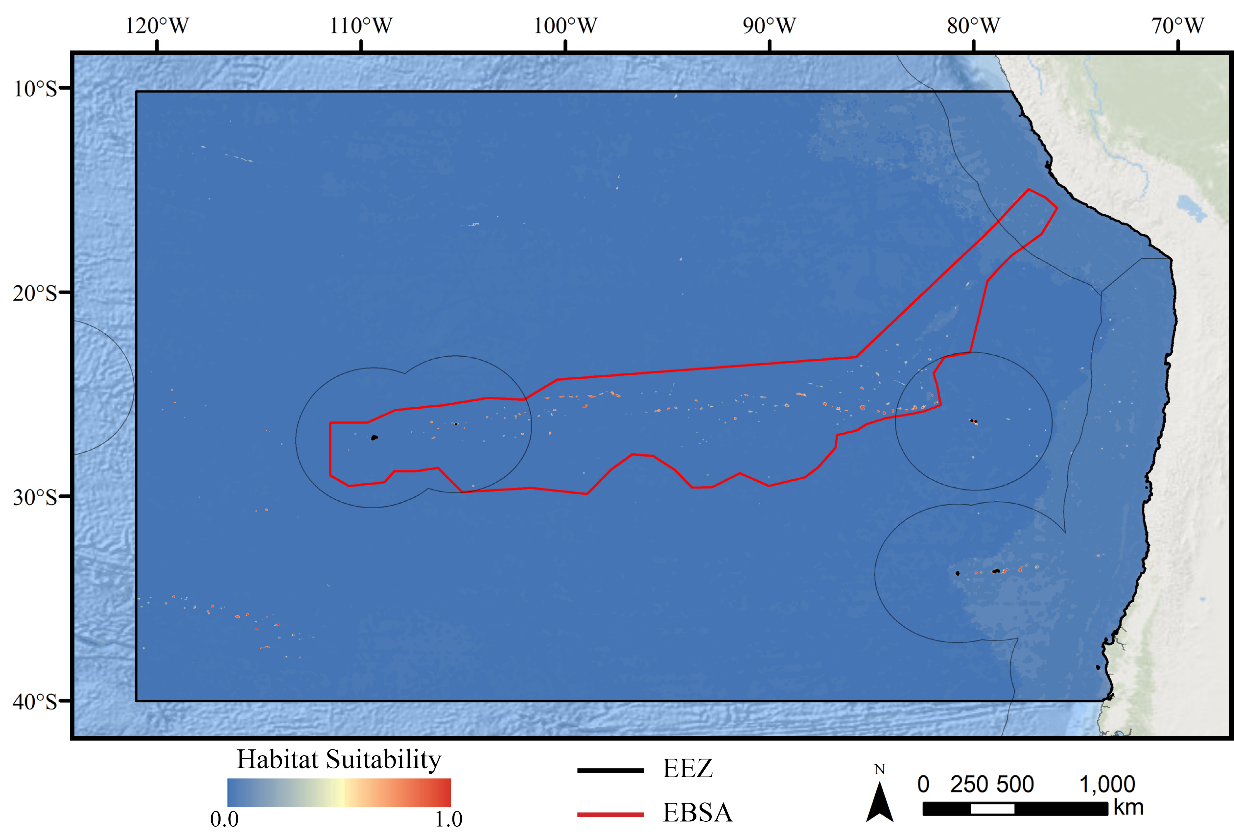

Supplement: Supplemental Information 14 — Warmer colors indicate more suitable habitat. [file peerj-09-11972-s014.png]

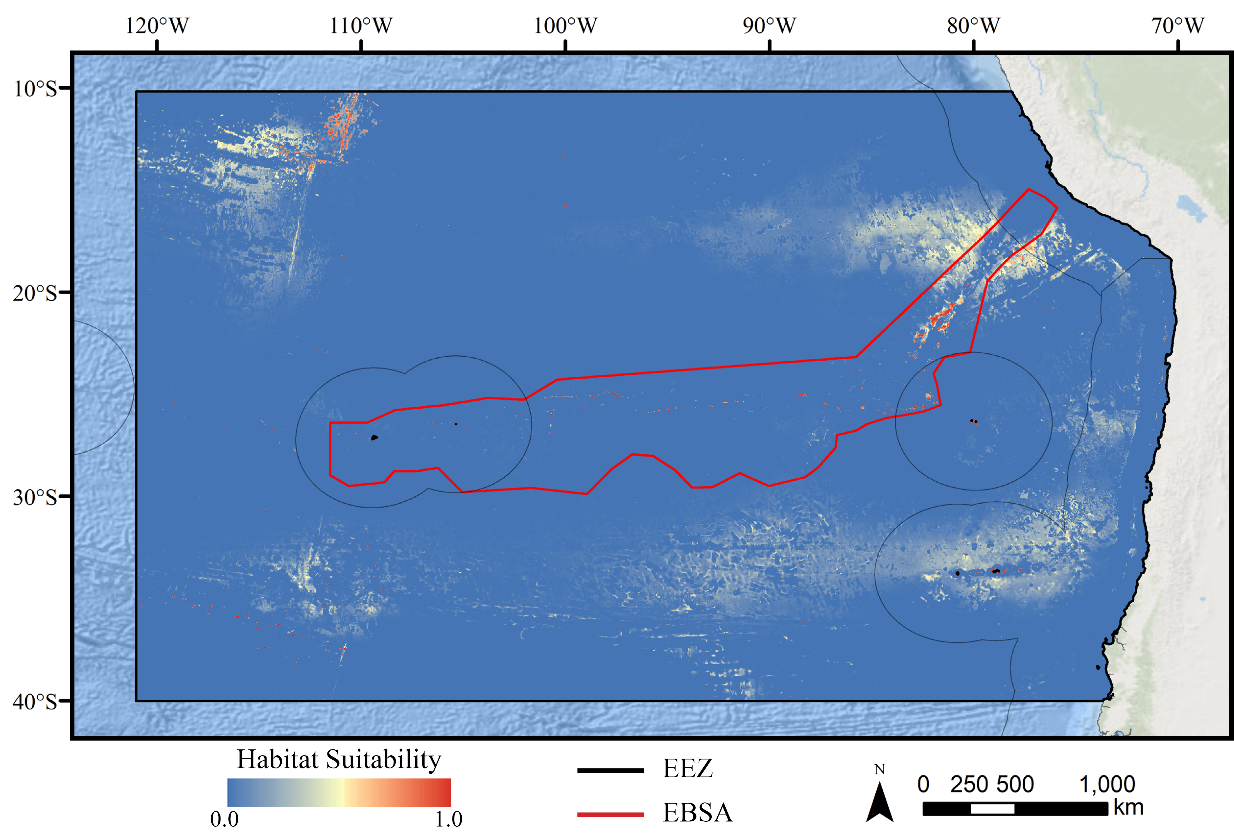

Supplement: Supplemental Information 15 — Warmer colors indicate more suitable habitat. [file peerj-09-11972-s015.png]

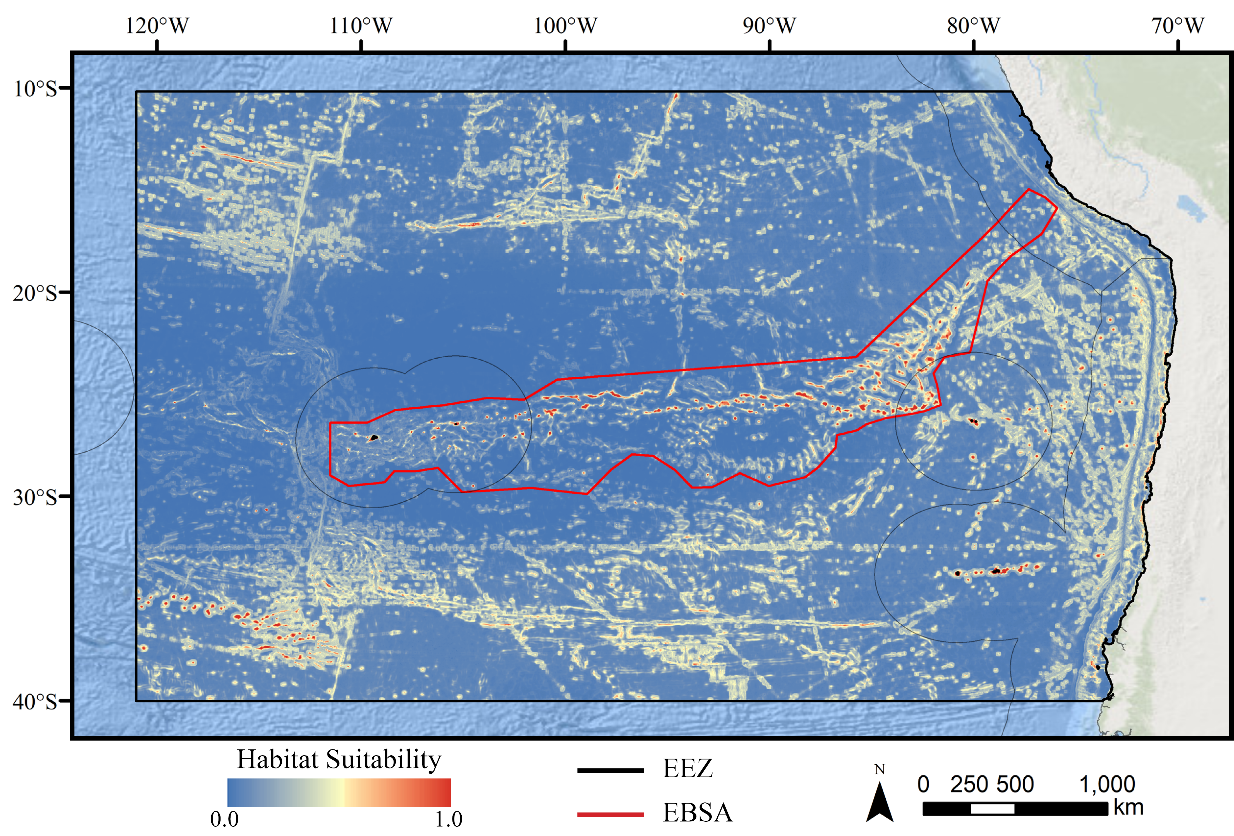

Supplement: Supplemental Information 16 — Warmer colors indicate more suitable habitat. [file peerj-09-11972-s016.png]

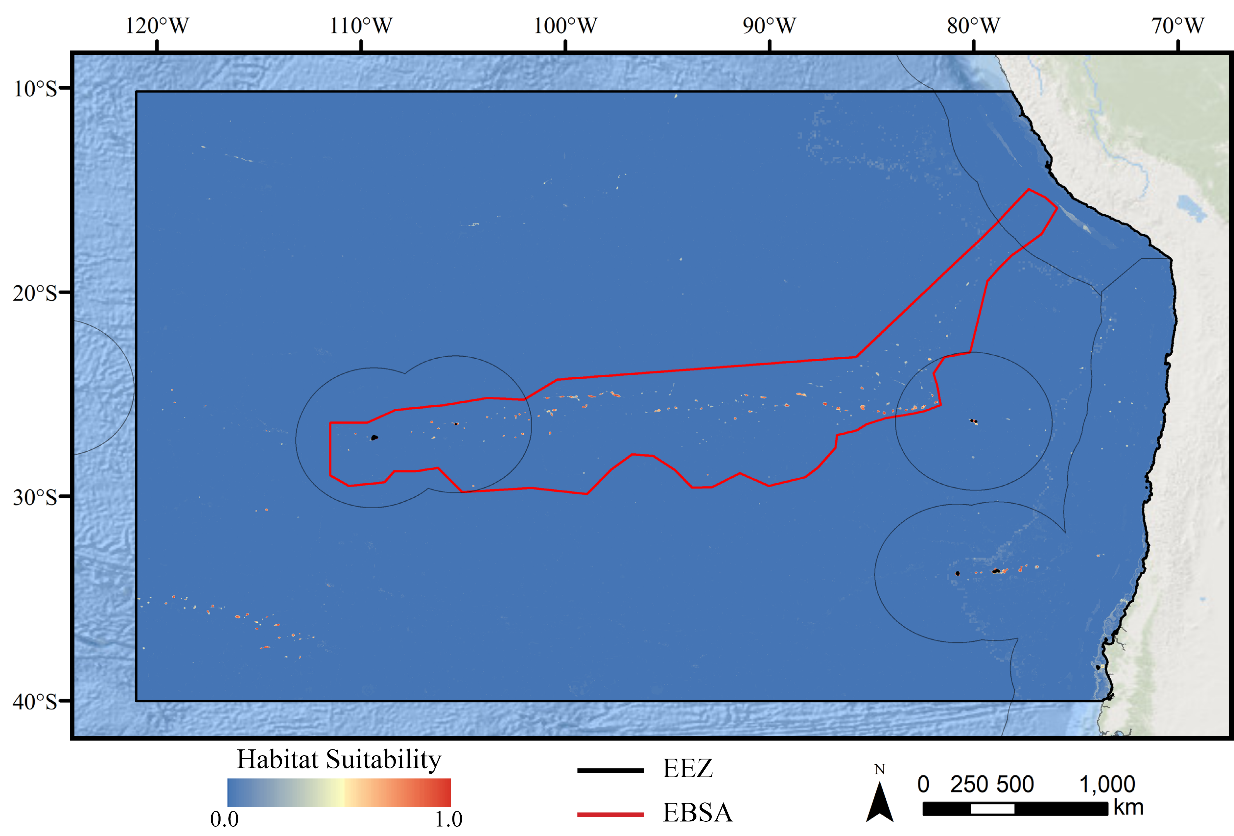

Supplement: Supplemental Information 17 — Warmer colors indicate more suitable habitat. [file peerj-09-11972-s017.png]

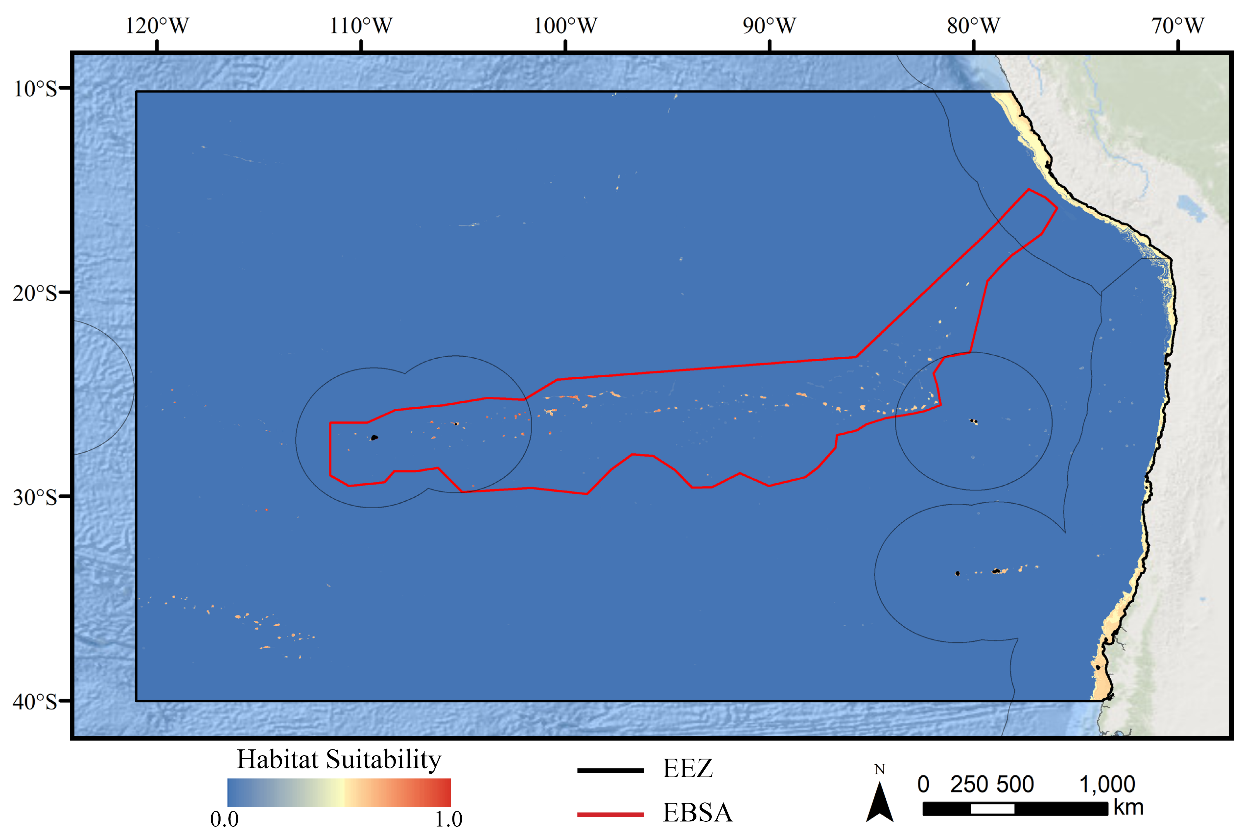

Supplement: Supplemental Information 18 — Warmer colors indicate more suitable habitat. [file peerj-09-11972-s018.png]

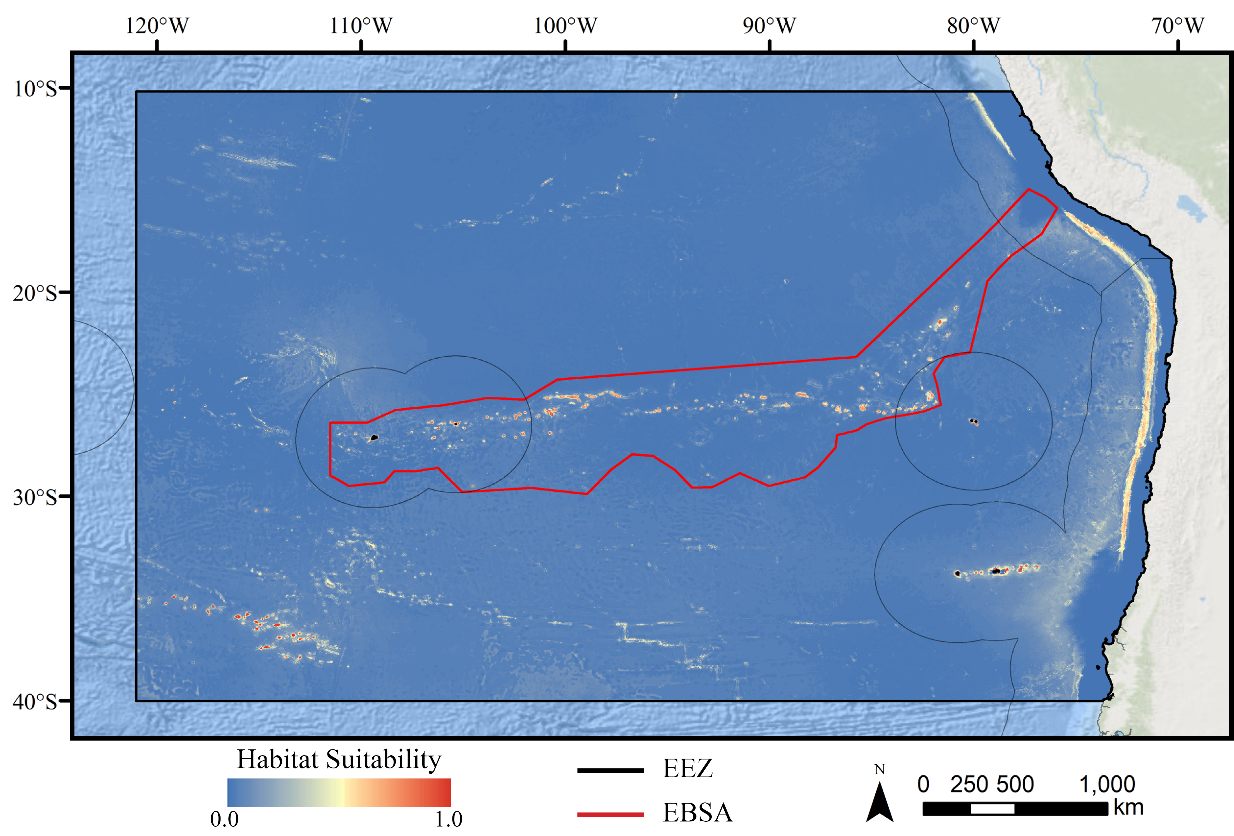

Supplement: Supplemental Information 19 — Warmer colors indicate more suitable habitat. [file peerj-09-11972-s019.png]

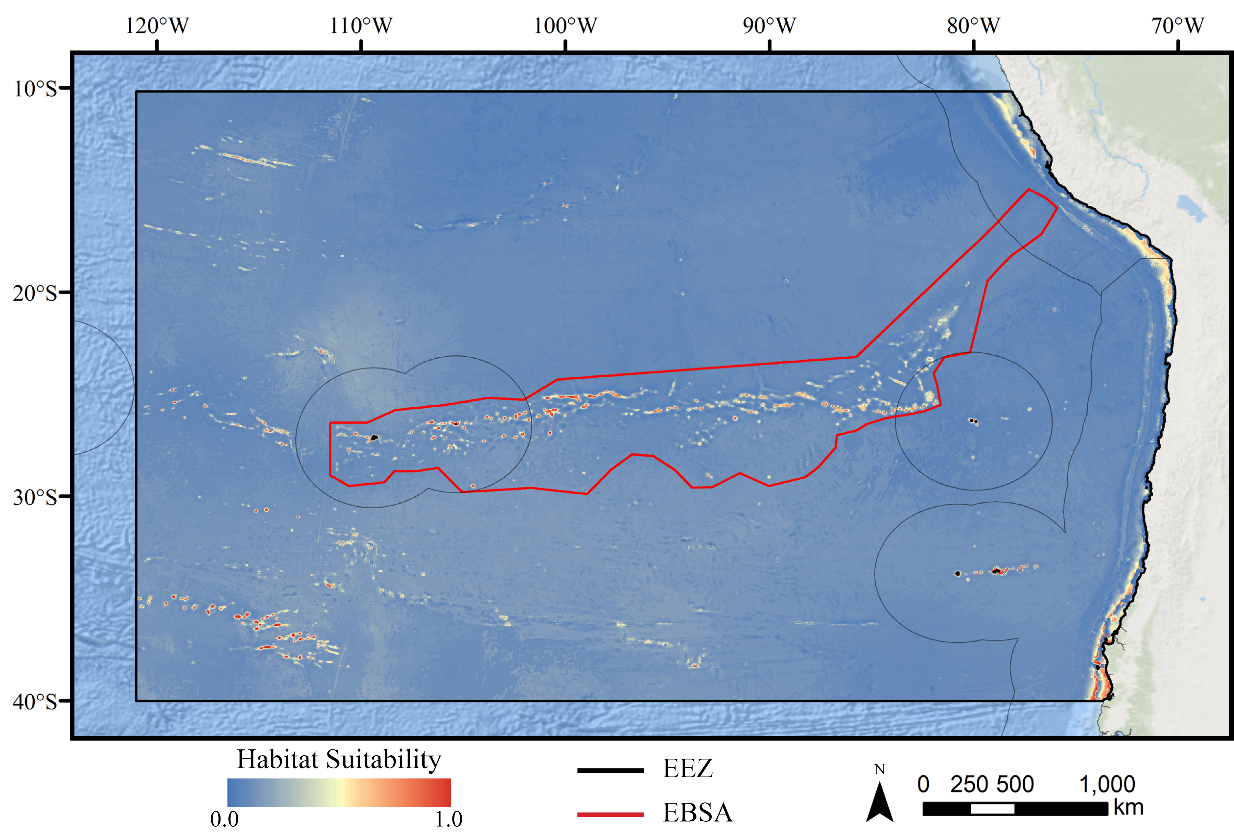

Supplement: Supplemental Information 20 — Warmer colors indicate more suitable habitat. [file peerj-09-11972-s020.png]

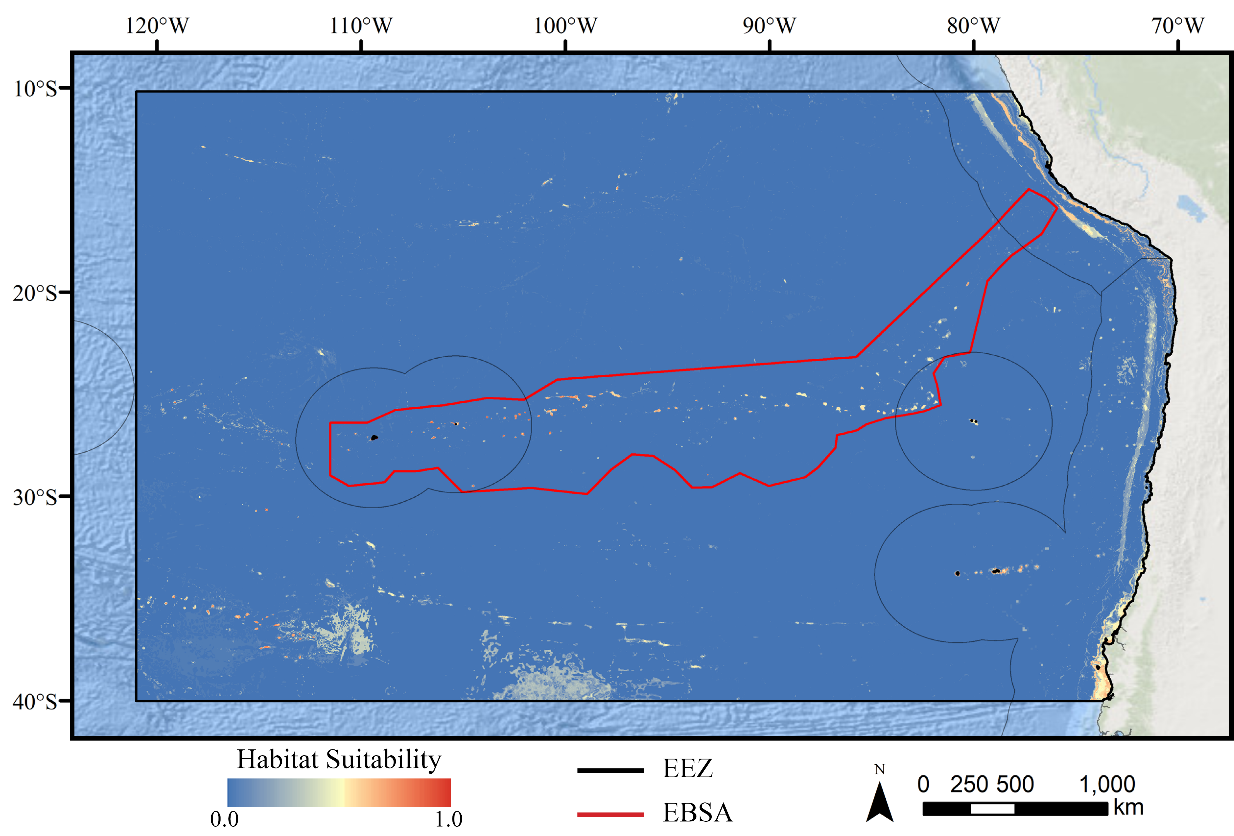

Supplement: Supplemental Information 21 — Warmer colors indicate more suitable habitat. [file peerj-09-11972-s021.png]

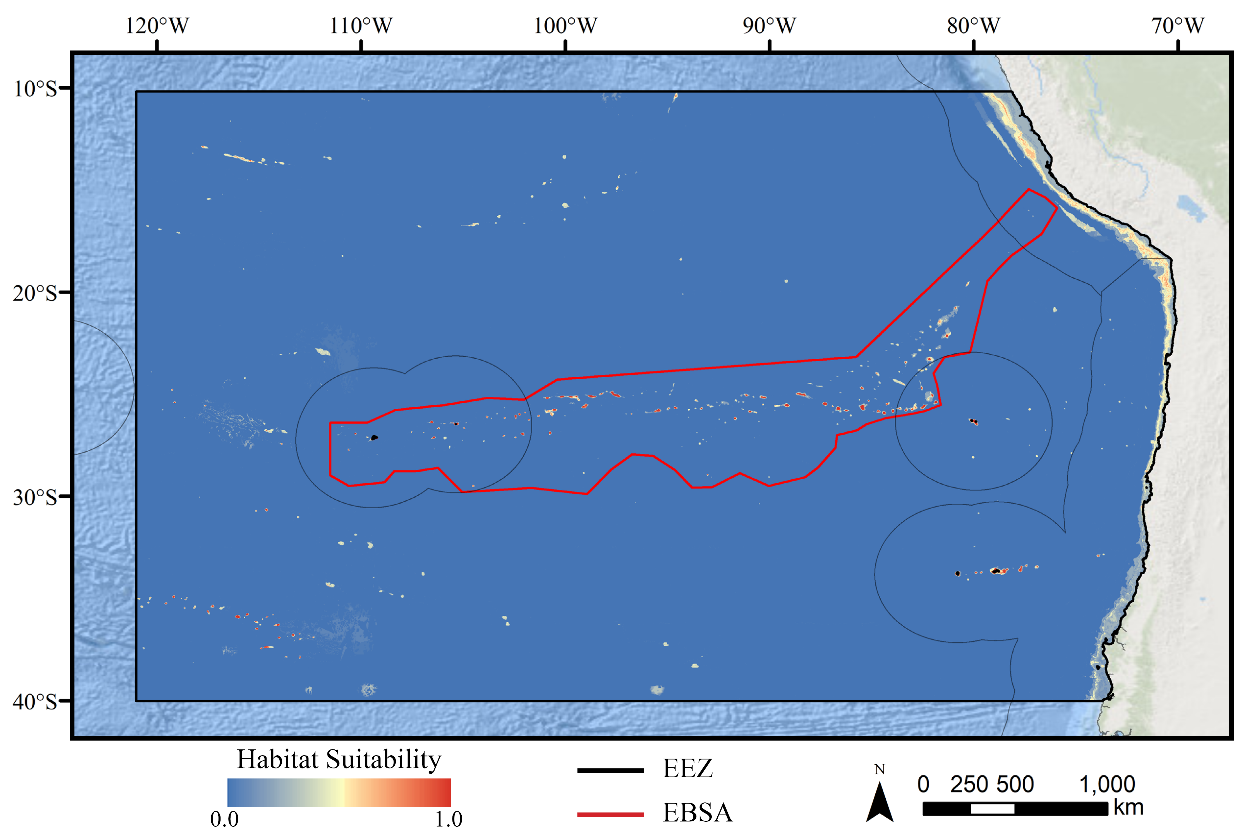

Supplement: Supplemental Information 22 — Warmer colors indicate more suitable habitat. [file peerj-09-11972-s022.png]

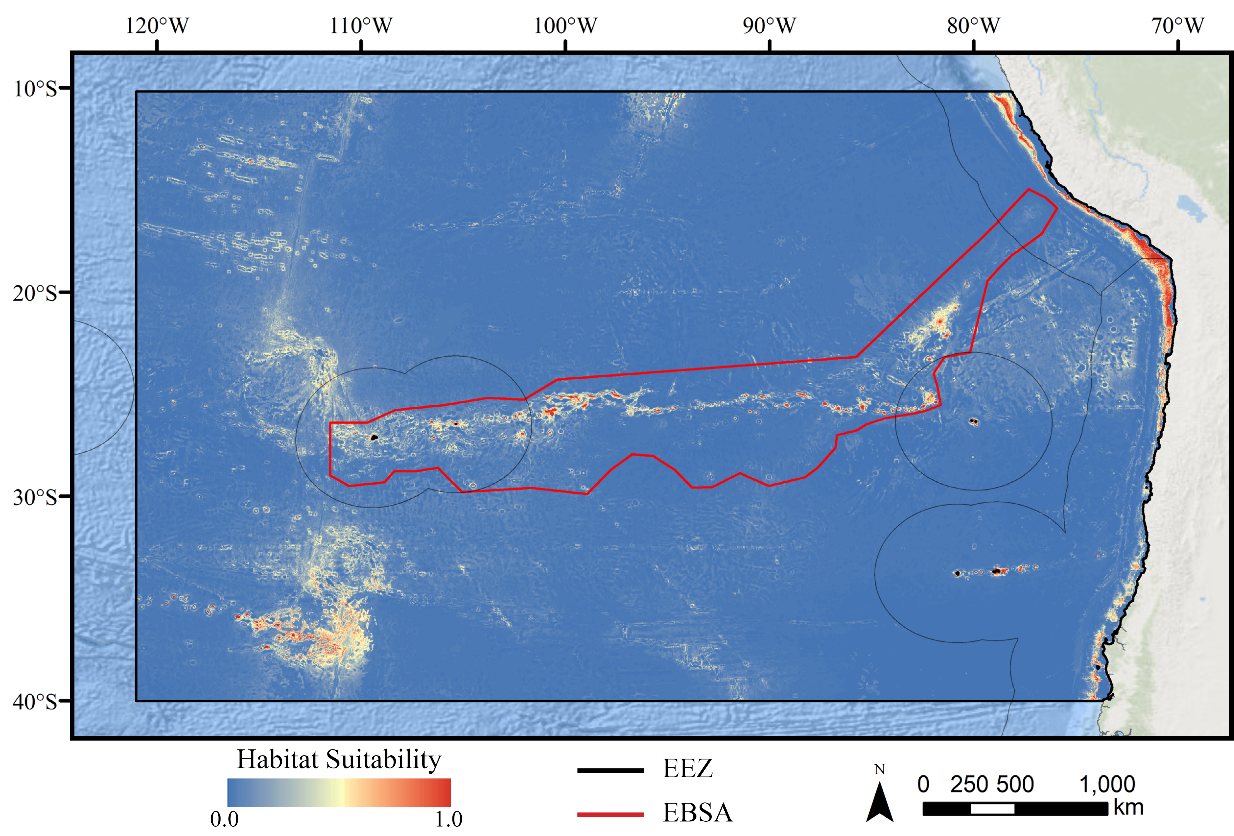

Supplement: Supplemental Information 23 — Warmer colors indicate more suitable habitat. [file peerj-09-11972-s023.png]

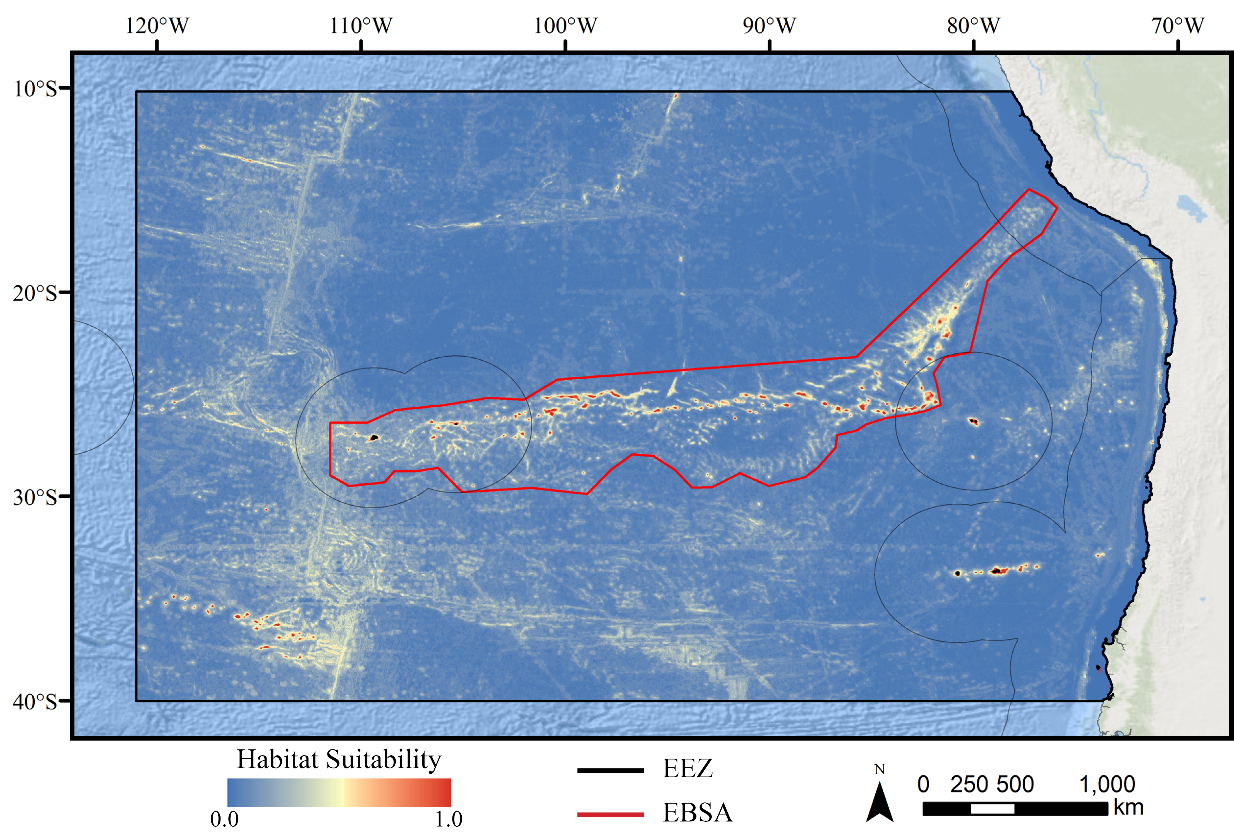

Supplement: Supplemental Information 24 — Warmer colors indicate more suitable habitat. [file peerj-09-11972-s024.png]

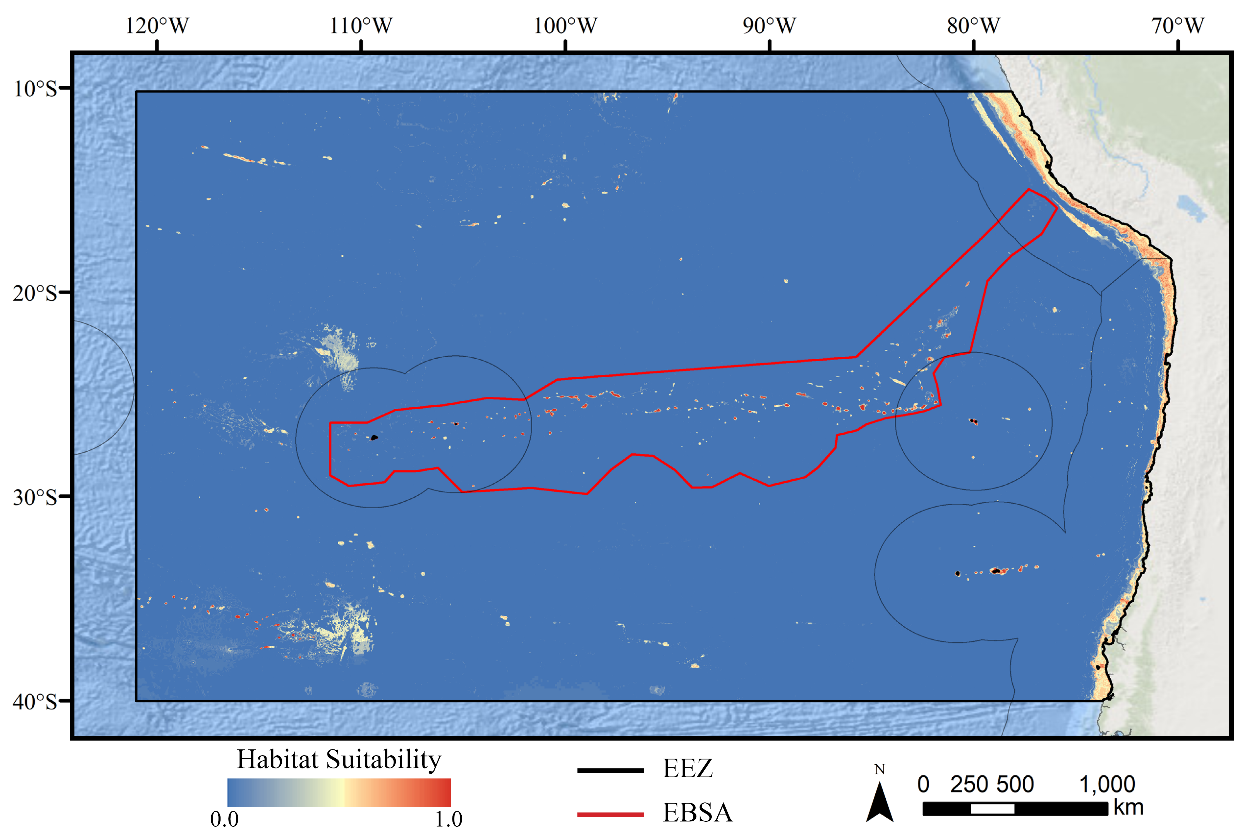

Supplement: Supplemental Information 25 — Warmer colors indicate more suitable habitat. [file peerj-09-11972-s025.png]

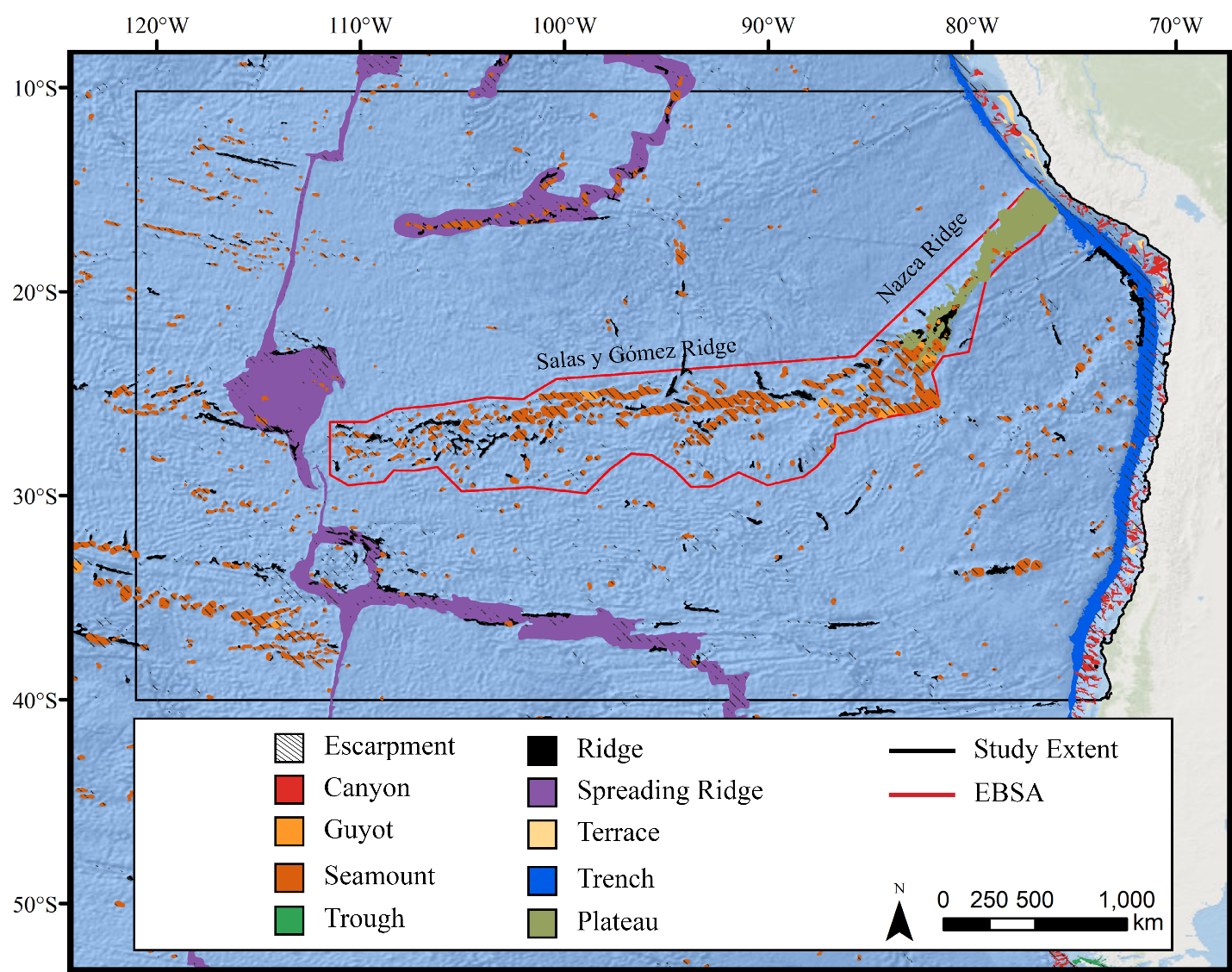

Supplement: Supplemental Information 26 [file peerj-09-11972-s026.png]

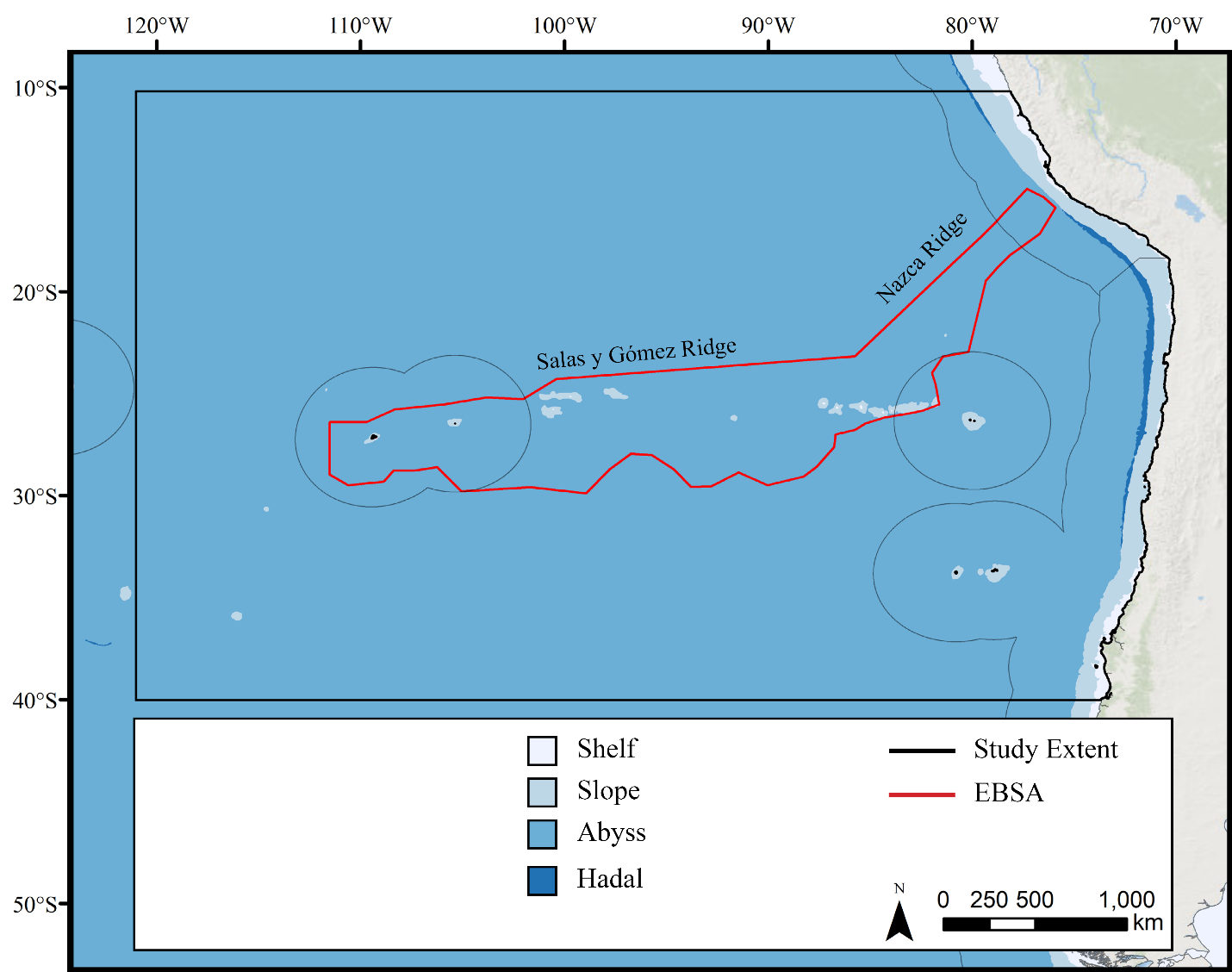

Supplement: Supplemental Information 27 [file peerj-09-11972-s027.png]

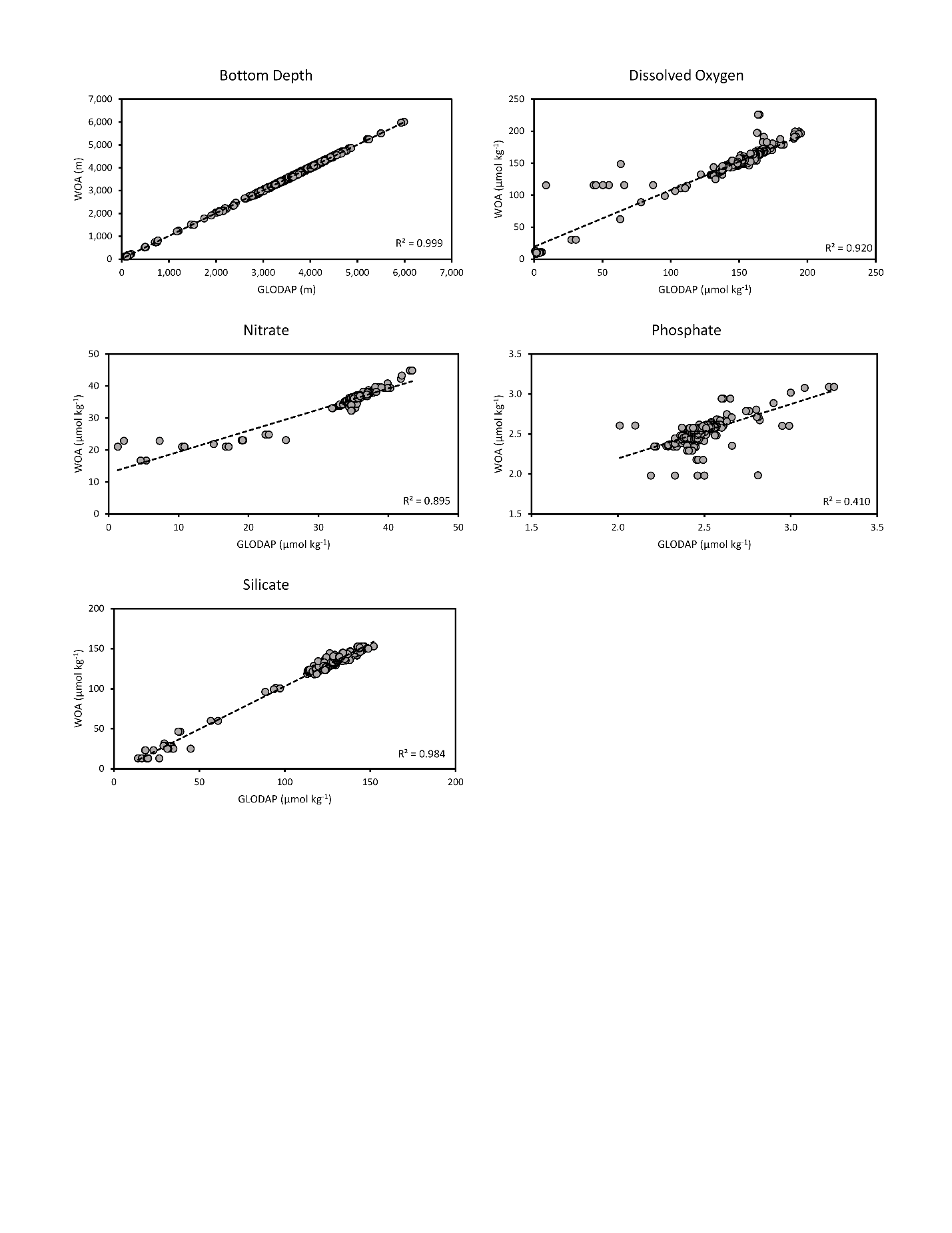

Supplement: Supplemental Information 28 — Each graph shows the correlation of the WOA datasets used during model creation with available quality-controlled bottle data from the Global Ocean Data Analysis Project (GLODAP v2.2019). Bottom-water data (within 50 m of the bottom) from both datasets were used. Bottom depth was not used in final models but is included here for reference. [file peerj-09-11972-s028.png]

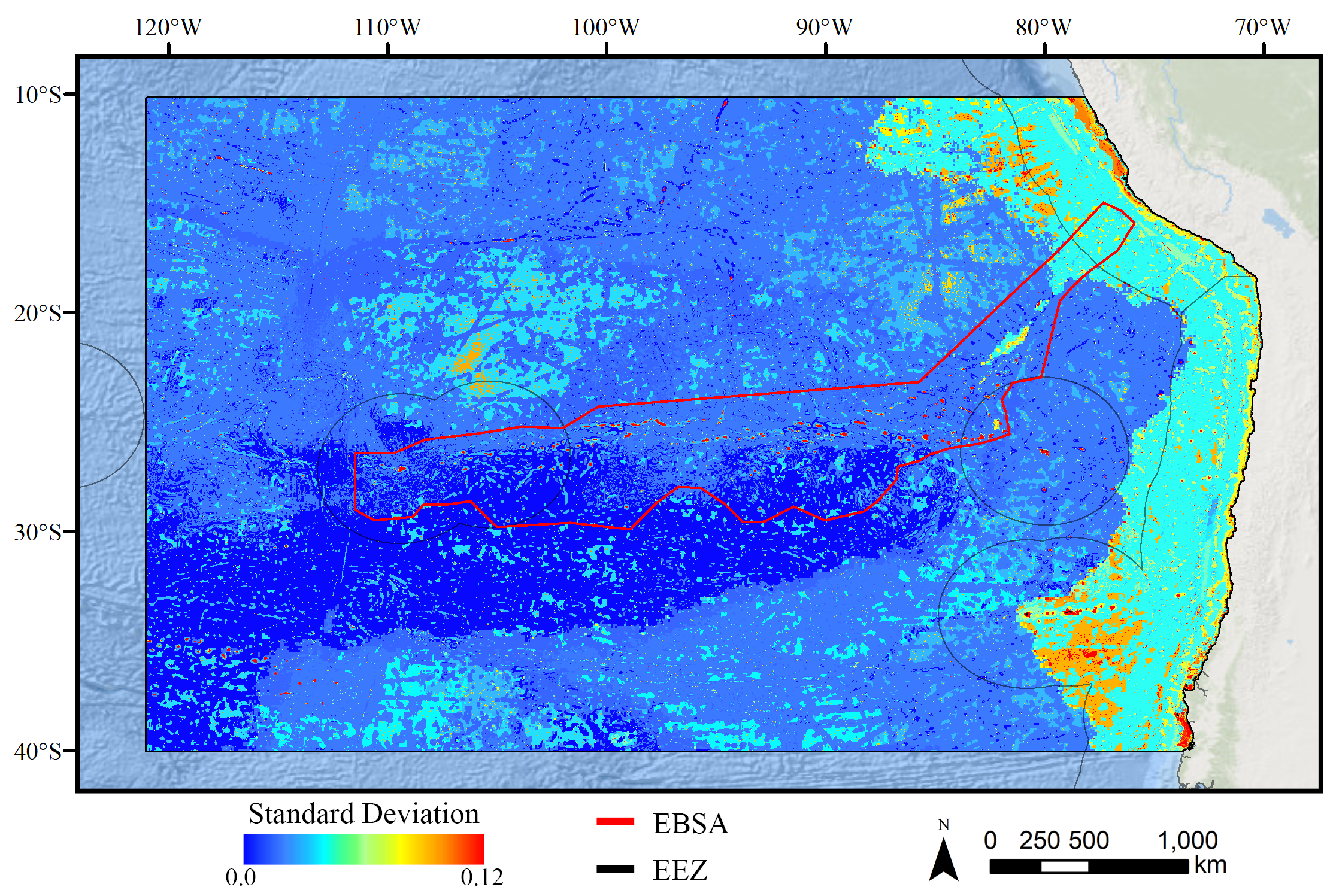

Supplement: Supplemental Information 29 — A ten-fold cross-validation procedure that randomly withheld 20% of occurrence and pseudoabsence data from model construction (with replacement between runs) was used to assess the spatial uncertainty of the predictions, shown here as the standard deviation of habitat suitability scores across all ten model runs. This approach does not account for all possible sources of uncertainty, but provides a useful spatial measure of how sensitive the model is to the sampling of occurrence data and the construction of the pseudoabsence dataset. Warmer colors indicate areas where model runs disagreed more prominently, indicating higher uncertainty in predictions. [file peerj-09-11972-s029.png]

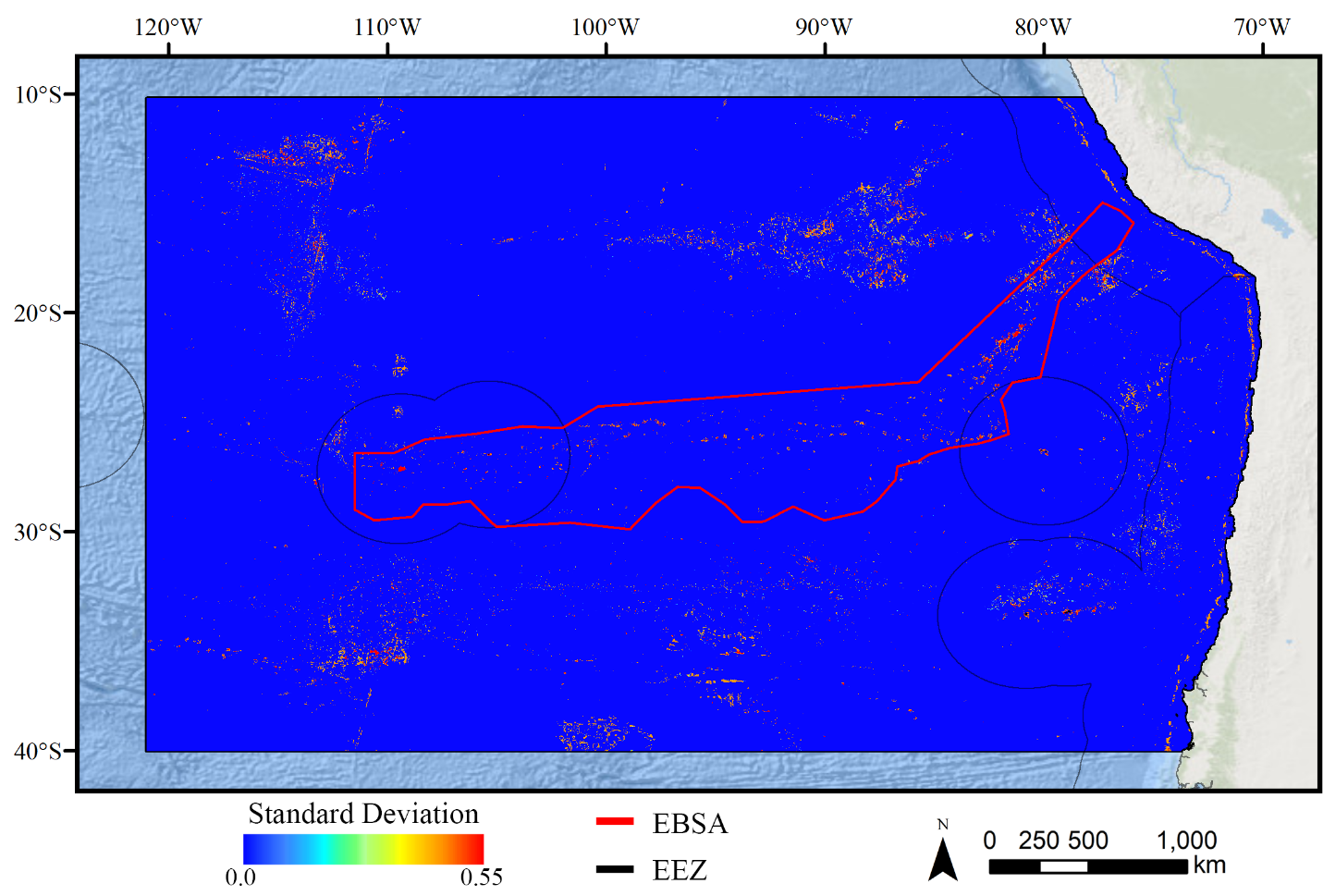

Supplement: Supplemental Information 30 — A ten-fold cross-validation procedure that randomly withheld 20% of occurrence and pseudoabsence data from model construction (with replacement between runs) was used to assess the spatial uncertainty of the predictions, shown here as the standard deviation of habitat suitability scores across all ten model runs. This approach does not account for all possible sources of uncertainty, but provides a useful spatial measure of how sensitive the model is to the sampling of occurrence data and the construction of the pseudoabsence dataset. Warmer colors indicate areas where model runs disagreed more prominently, indicating higher uncertainty in predictions. [file peerj-09-11972-s030.png]

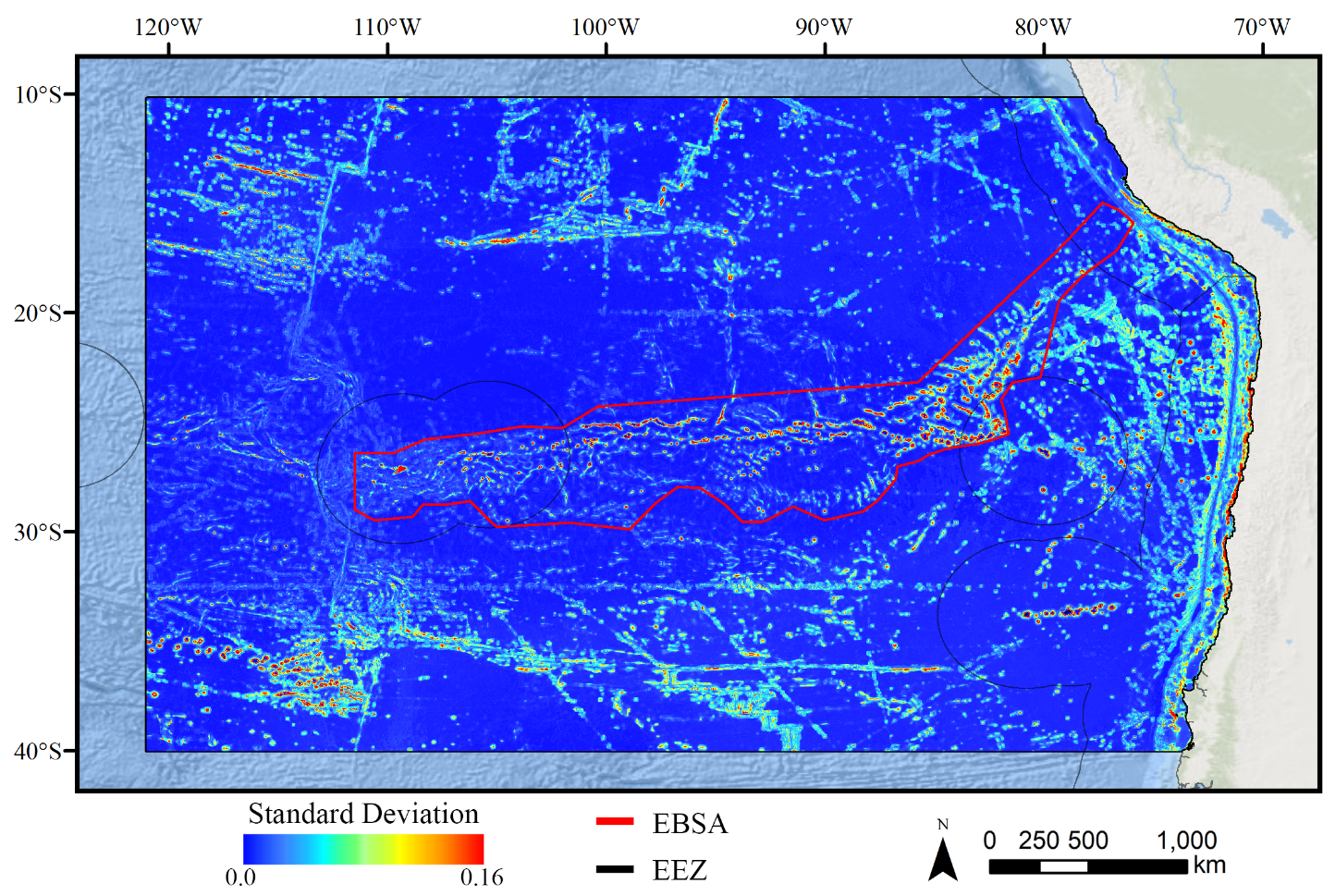

Supplement: Supplemental Information 31 — A ten-fold cross-validation procedure that randomly withheld 20% of occurrence and pseudoabsence data from model construction (with replacement between runs) was used to assess the spatial uncertainty of the predictions, shown here as the standard deviation of habitat suitability scores across all ten model runs. This approach does not account for all possible sources of uncertainty, but provides a useful spatial measure of how sensitive the model is to the sampling of occurrence data and the construction of the pseudoabsence dataset. Warmer colors indicate areas where model runs disagreed more prominently, indicating higher uncertainty in predictions. [file peerj-09-11972-s031.png]

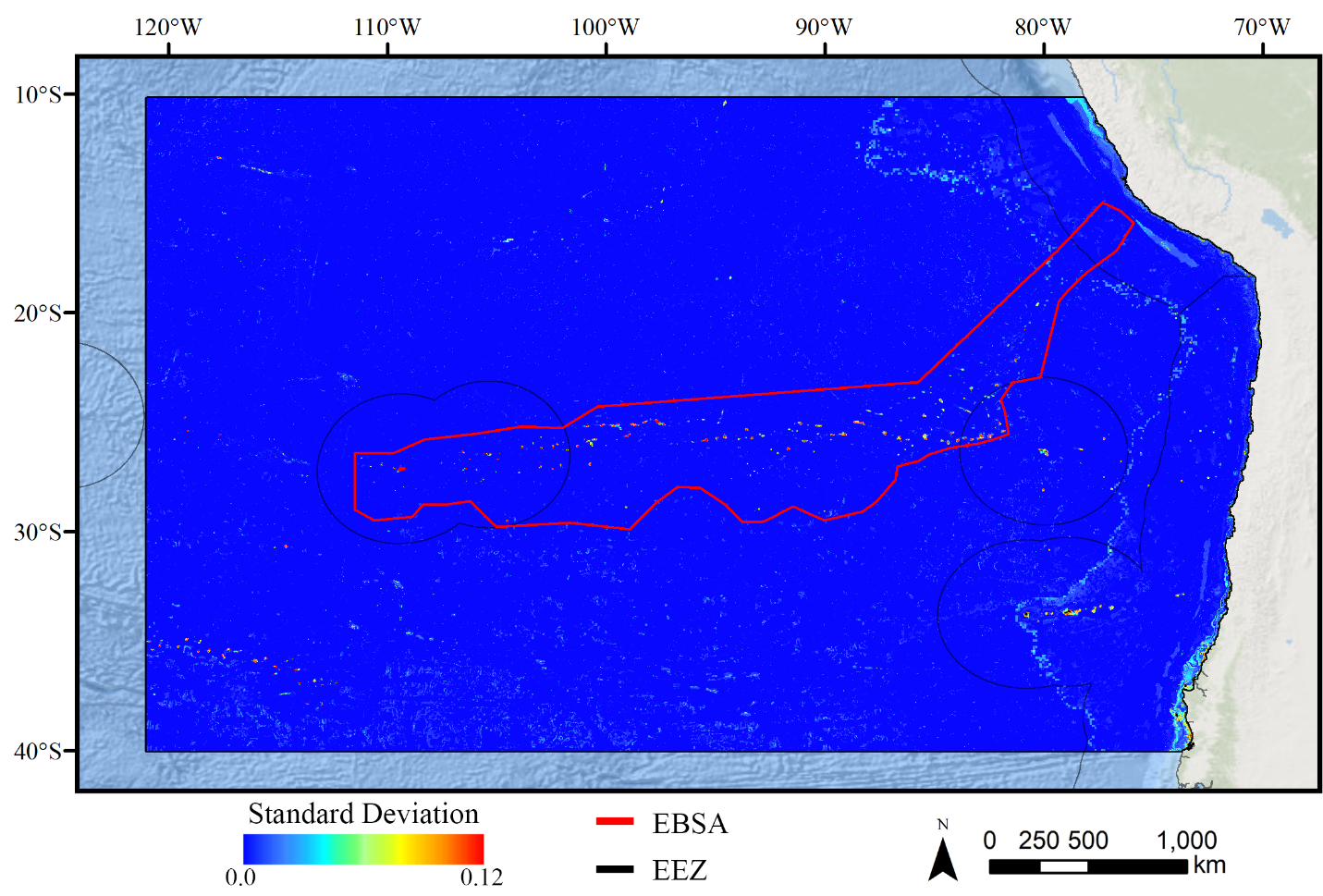

Supplement: Supplemental Information 32 — A ten-fold cross-validation procedure that randomly withheld 20% of occurrence and pseudoabsence data from model construction (with replacement between runs) was used to assess the spatial uncertainty of the predictions, shown here as the standard deviation of habitat suitability scores across all ten model runs. This approach does not account for all possible sources of uncertainty, but provides a useful spatial measure of how sensitive the model is to the sampling of occurrence data and the construction of the pseudoabsence dataset. Warmer colors indicate areas where model runs disagreed more prominently, indicating higher uncertainty in predictions. [file peerj-09-11972-s032.png]

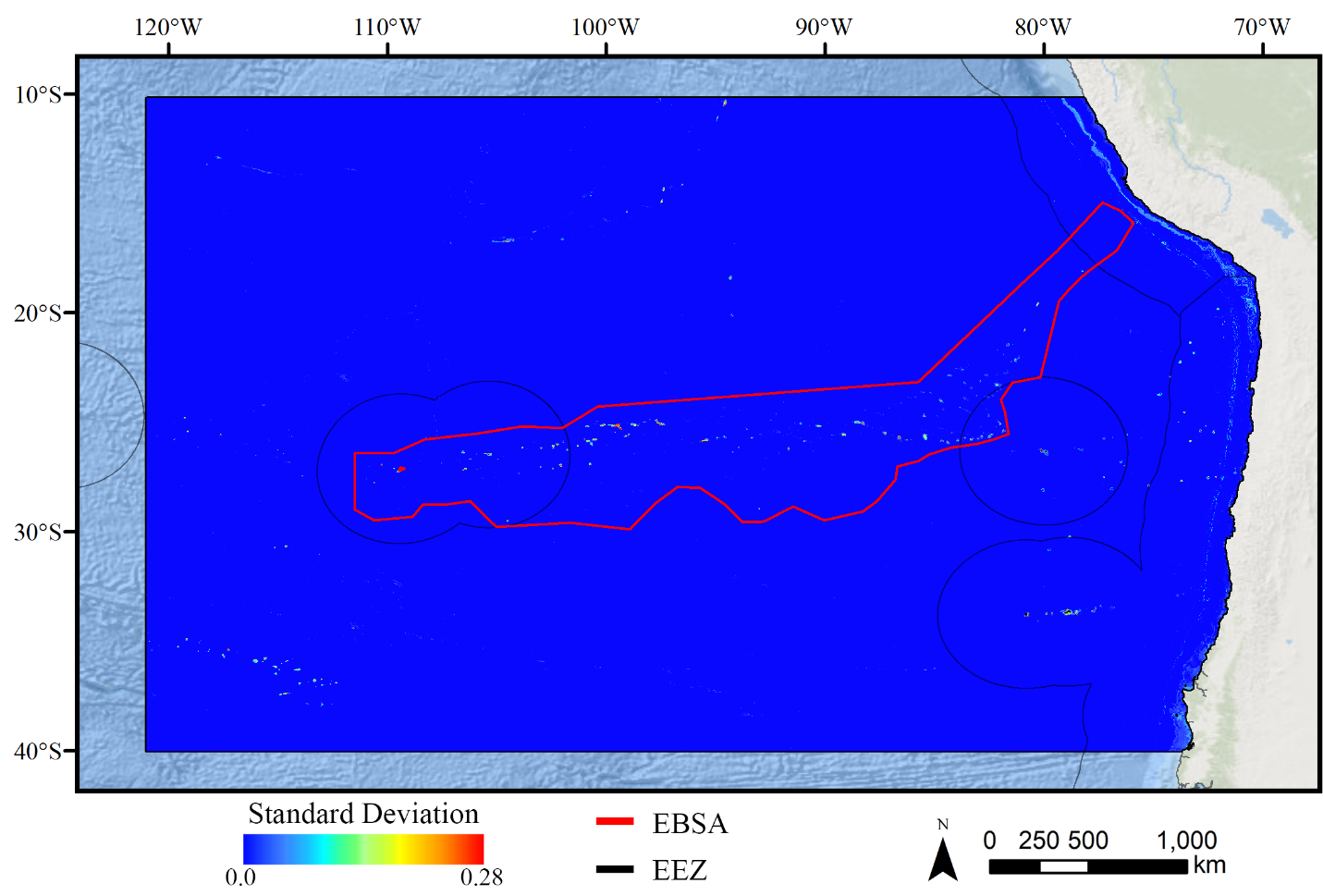

Supplement: Supplemental Information 33 — A ten-fold cross-validation procedure that randomly withheld 20% of occurrence and pseudoabsence data from model construction (with replacement between runs) was used to assess the spatial uncertainty of the predictions, shown here as the standard deviation of habitat suitability scores across all ten model runs. This approach does not account for all possible sources of uncertainty, but provides a useful spatial measure of how sensitive the model is to the sampling of occurrence data and the construction of the pseudoabsence dataset. Warmer colors indicate areas where model runs disagreed more prominently, indicating higher uncertainty in predictions. [file peerj-09-11972-s033.png]

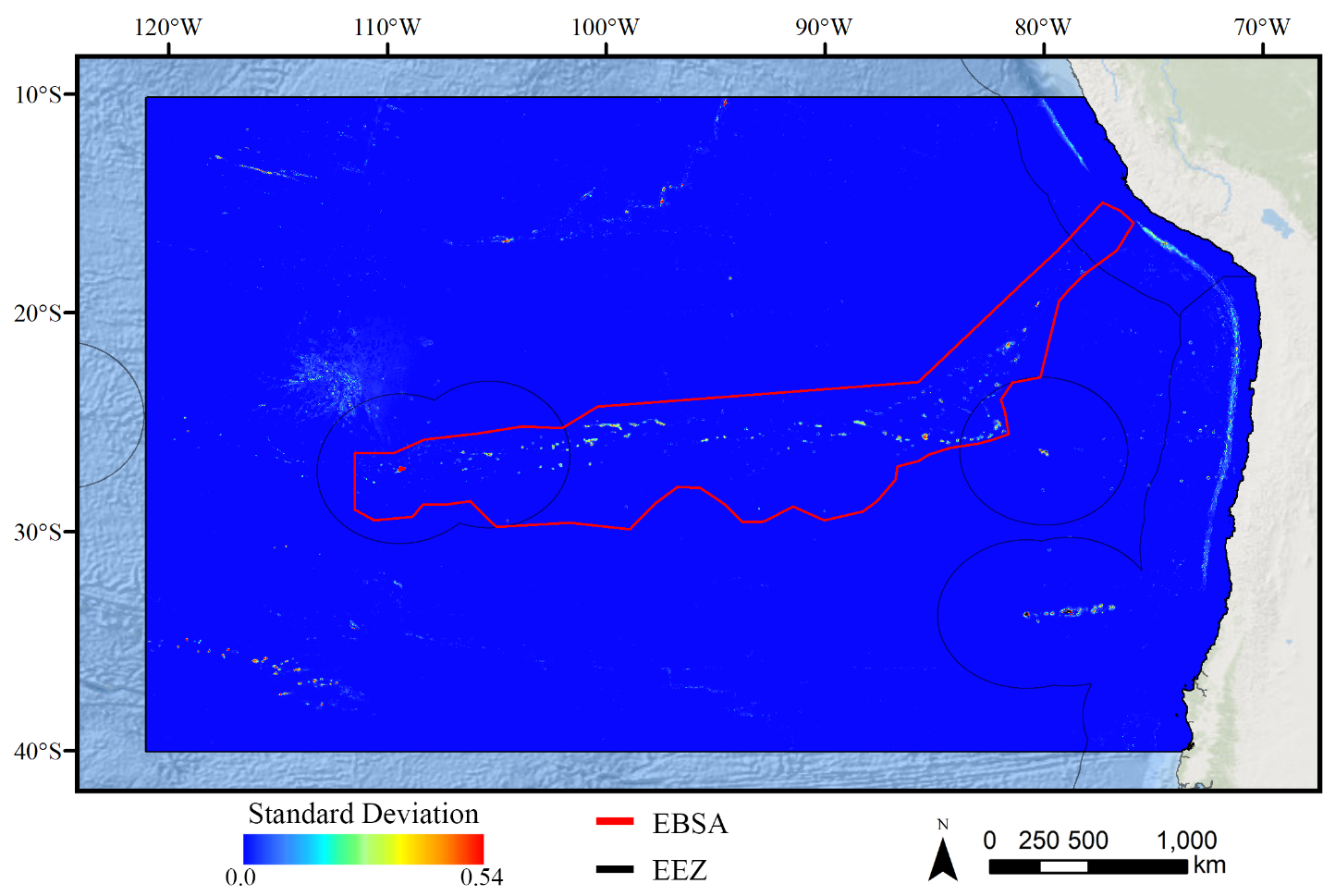

Supplement: Supplemental Information 34 — A ten-fold cross-validation procedure that randomly withheld 20% of occurrence and pseudoabsence data from model construction (with replacement between runs) was used to assess the spatial uncertainty of the predictions, shown here as the standard deviation of habitat suitability scores across all ten model runs. This approach does not account for all possible sources of uncertainty, but provides a useful spatial measure of how sensitive the model is to the sampling of occurrence data and the construction of the pseudoabsence dataset. Warmer colors indicate areas where model runs disagreed more prominently, indicating higher uncertainty in predictions. [file peerj-09-11972-s034.png]

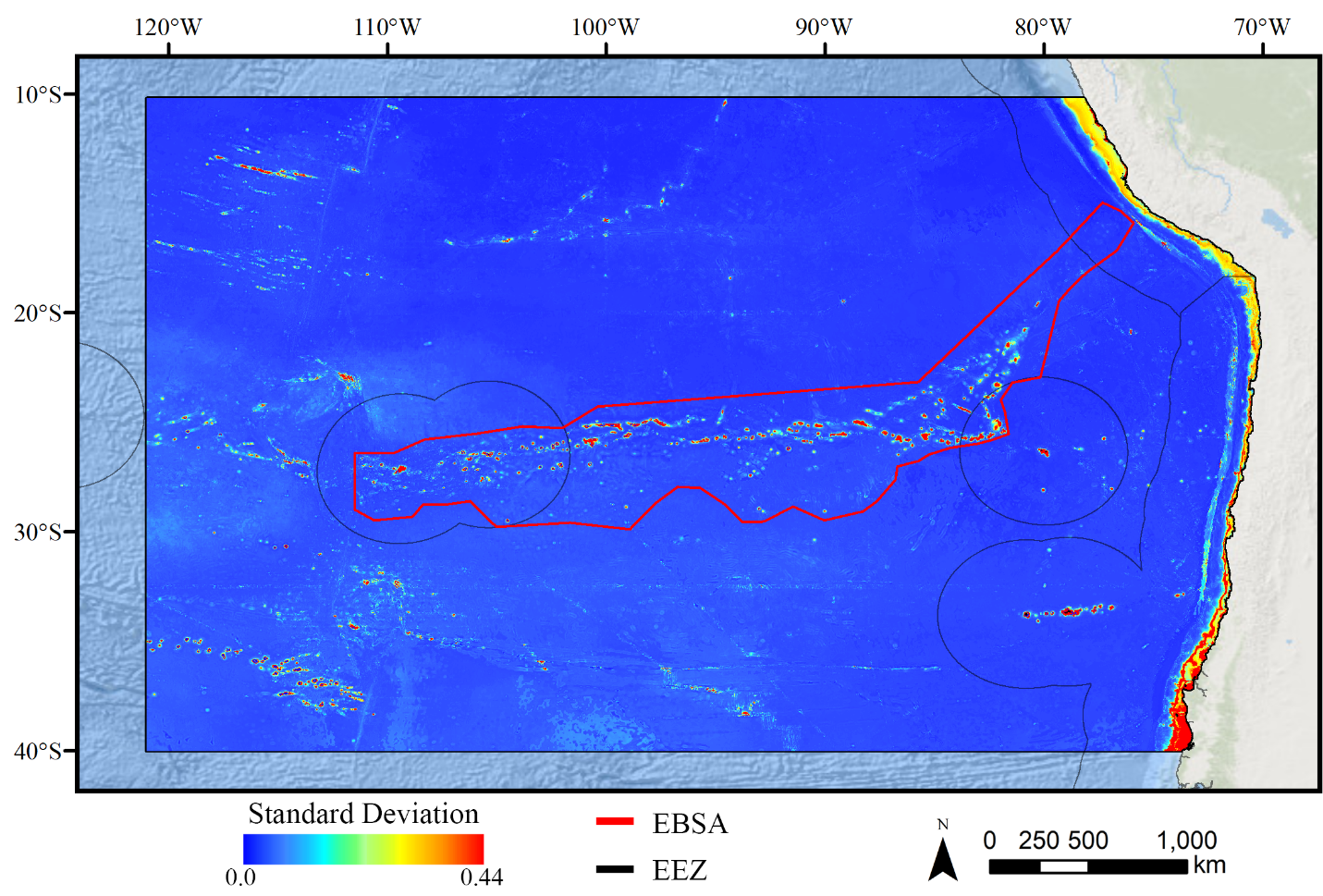

Supplement: Supplemental Information 35 — A ten-fold cross-validation procedure that randomly withheld 20% of occurrence and pseudoabsence data from model construction (with replacement between runs) was used to assess the spatial uncertainty of the predictions, shown here as the standard deviation of habitat suitability scores across all ten model runs. This approach does not account for all possible sources of uncertainty, but provides a useful spatial measure of how sensitive the model is to the sampling of occurrence data and the construction of the pseudoabsence dataset. Warmer colors indicate areas where model runs disagreed more prominently, indicating higher uncertainty in predictions. [file peerj-09-11972-s035.png]

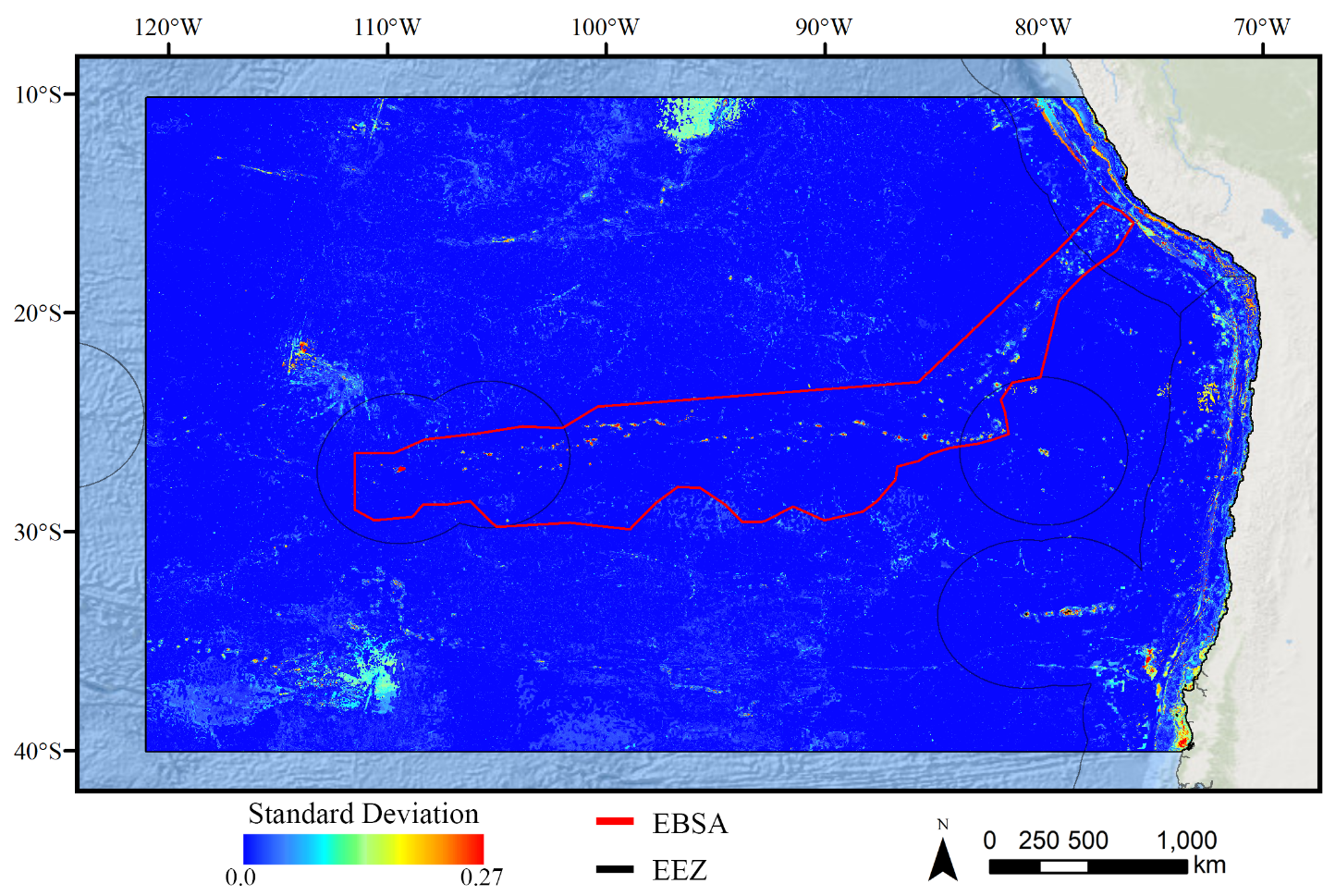

Supplement: Supplemental Information 36 — A ten-fold cross-validation procedure that randomly withheld 20% of occurrence and pseudoabsence data from model construction (with replacement between runs) was used to assess the spatial uncertainty of the predictions, shown here as the standard deviation of habitat suitability scores across all ten model runs. This approach does not account for all possible sources of uncertainty, but provides a useful spatial measure of how sensitive the model is to the sampling of occurrence data and the construction of the pseudoabsence dataset. Warmer colors indicate areas where model runs disagreed more prominently, indicating higher uncertainty in predictions. [file peerj-09-11972-s036.png]

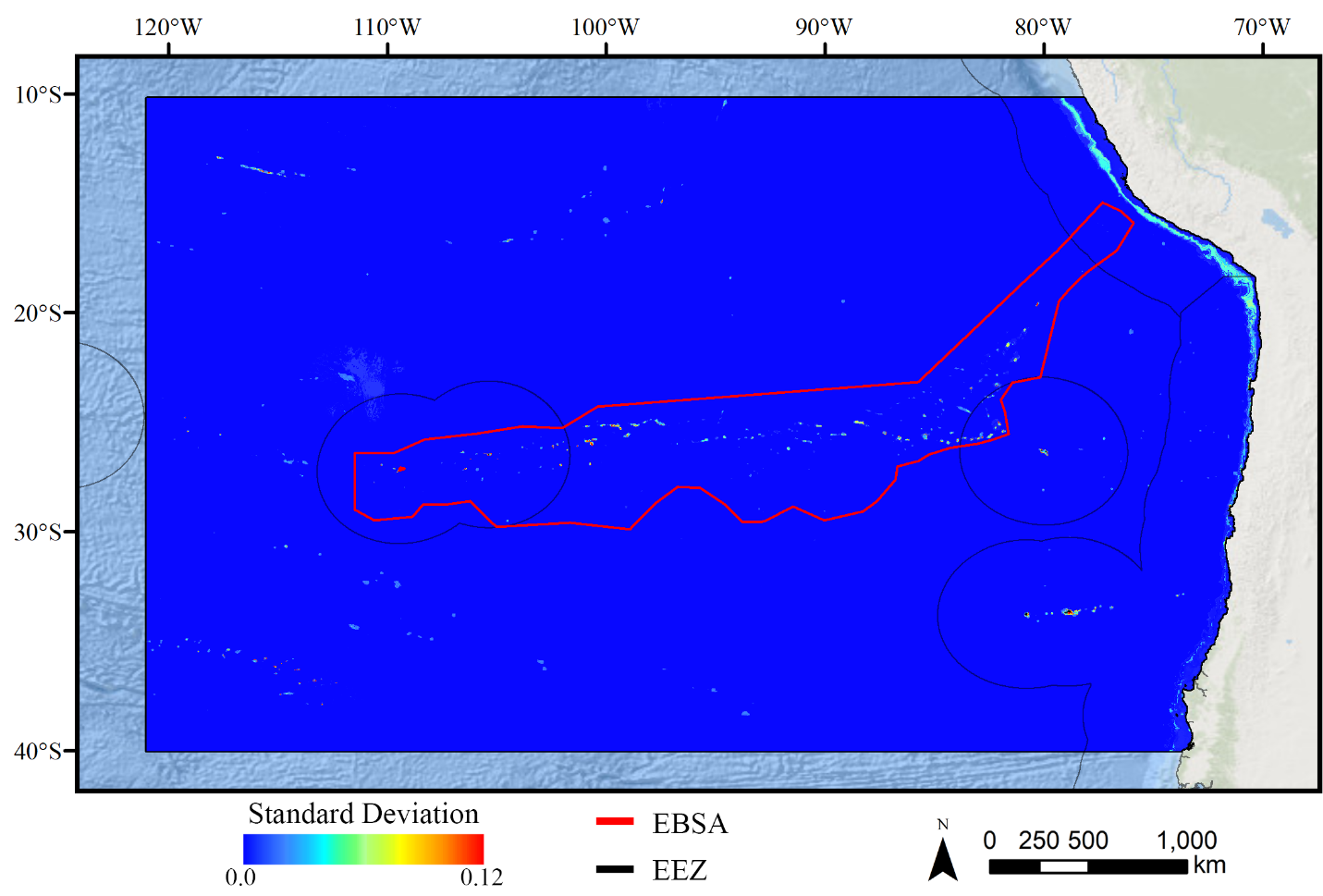

Supplement: Supplemental Information 37 — A ten-fold cross-validation procedure that randomly withheld 20% of occurrence and pseudoabsence data from model construction (with replacement between runs) was used to assess the spatial uncertainty of the predictions, shown here as the standard deviation of habitat suitability scores across all ten model runs. This approach does not account for all possible sources of uncertainty, but provides a useful spatial measure of how sensitive the model is to the sampling of occurrence data and the construction of the pseudoabsence dataset. Warmer colors indicate areas where model runs disagreed more prominently, indicating higher uncertainty in predictions. [file peerj-09-11972-s037.png]

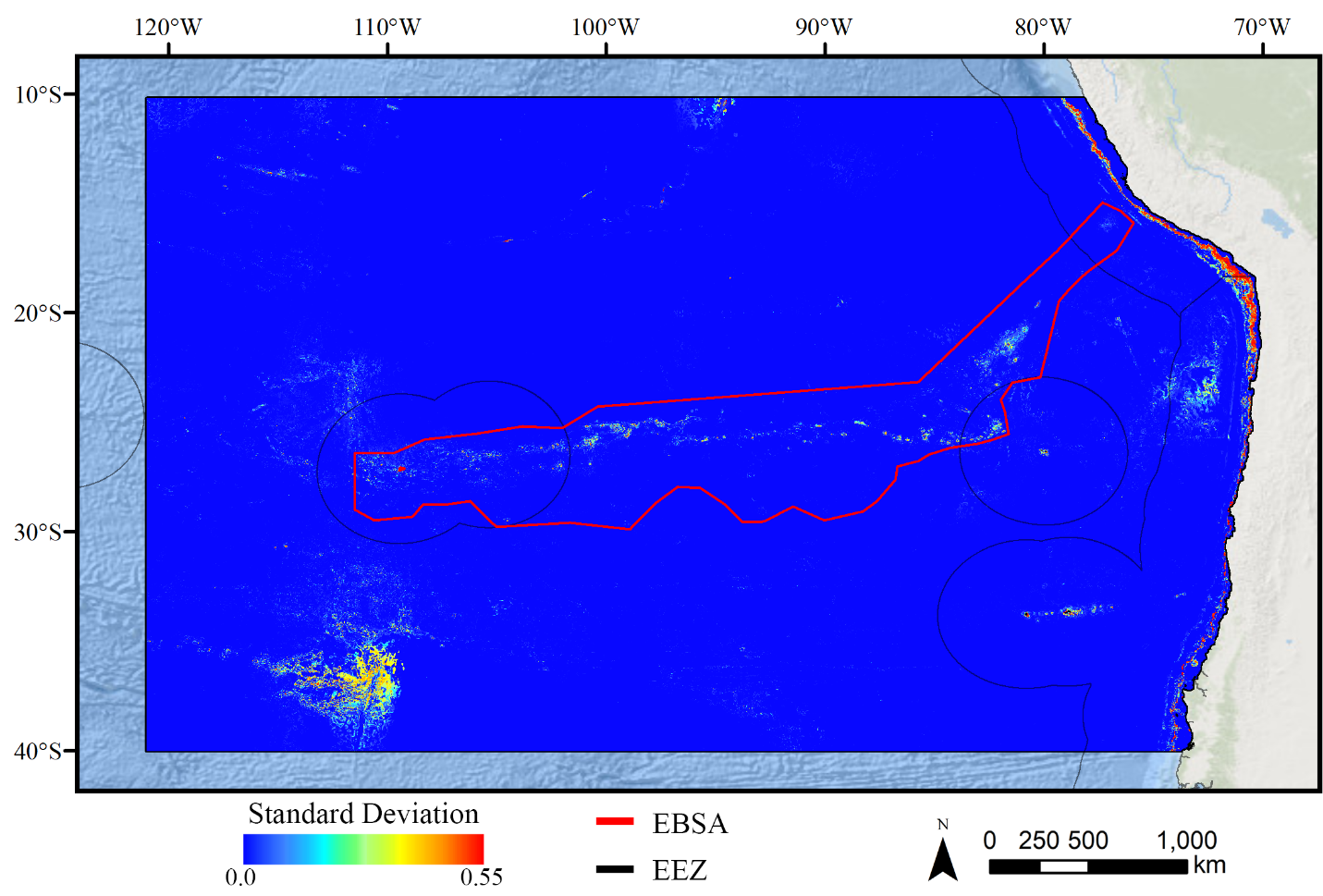

Supplement: Supplemental Information 38 — A ten-fold cross-validation procedure that randomly withheld 20% of occurrence and pseudoabsence data from model construction (with replacement between runs) was used to assess the spatial uncertainty of the predictions, shown here as the standard deviation of habitat suitability scores across all ten model runs. This approach does not account for all possible sources of uncertainty, but provides a useful spatial measure of how sensitive the model is to the sampling of occurrence data and the construction of the pseudoabsence dataset. Warmer colors indicate areas where model runs disagreed more prominently, indicating higher uncertainty in predictions. [file peerj-09-11972-s038.png]

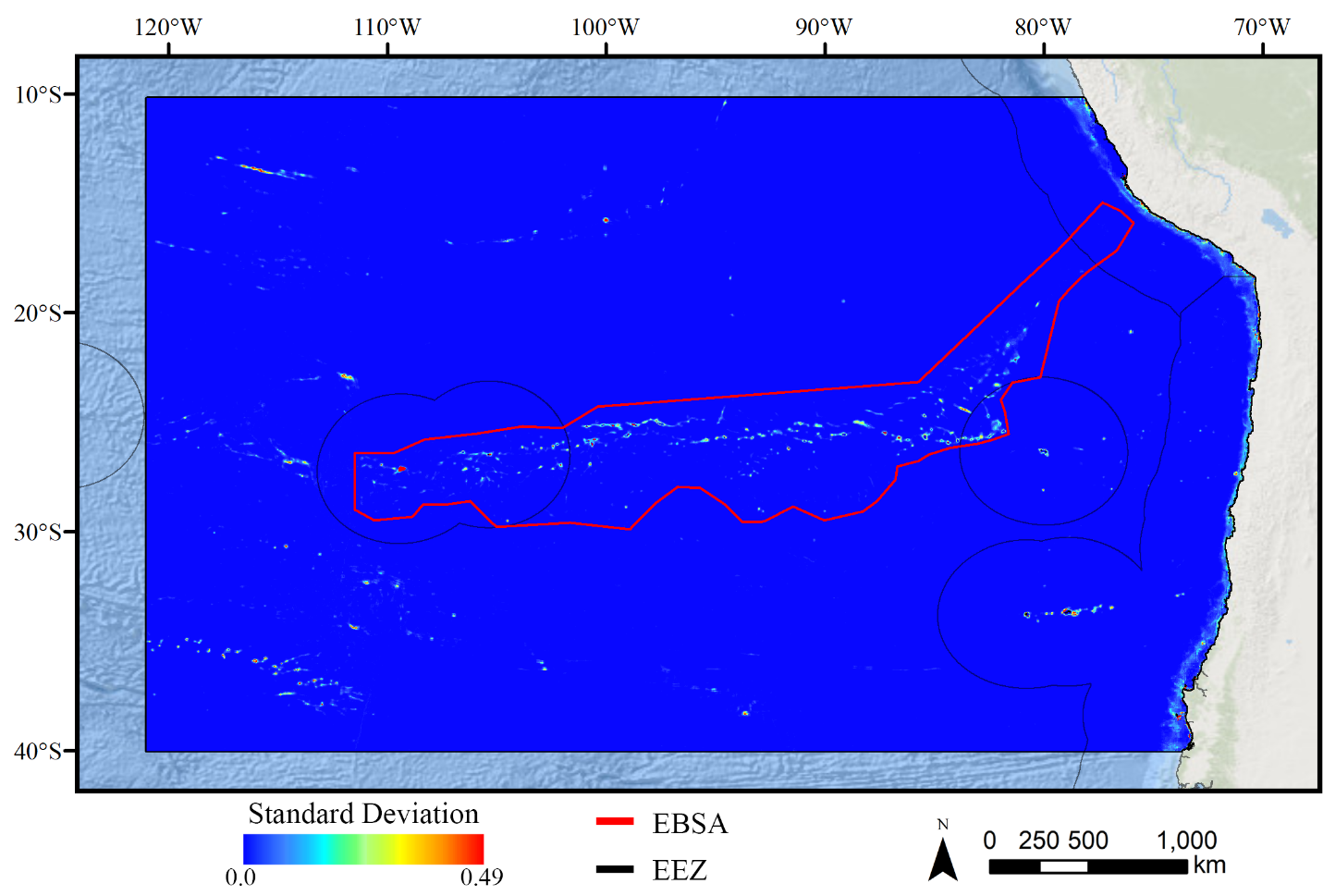

Supplement: Supplemental Information 39 — A ten-fold cross-validation procedure that randomly withheld 20% of occurrence and pseudoabsence data from model construction (with replacement between runs) was used to assess the spatial uncertainty of the predictions, shown here as the standard deviation of habitat suitability scores across all ten model runs. This approach does not account for all possible sources of uncertainty, but provides a useful spatial measure of how sensitive the model is to the sampling of occurrence data and the construction of the pseudoabsence dataset. Warmer colors indicate areas where model runs disagreed more prominently, indicating higher uncertainty in predictions. [file peerj-09-11972-s039.png]

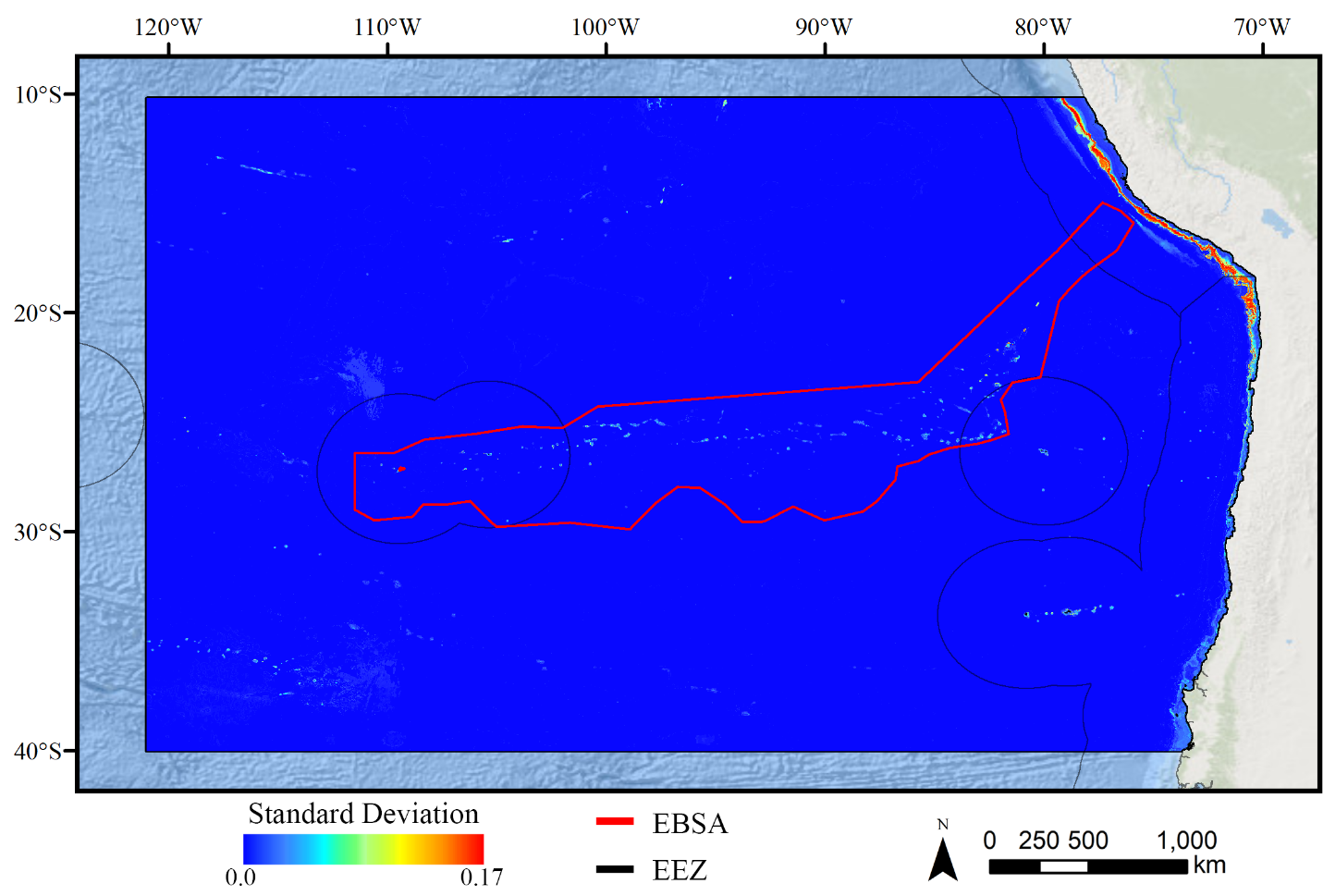

Supplement: Supplemental Information 40 — A ten-fold cross-validation procedure that randomly withheld 20% of occurrence and pseudoabsence data from model construction (with replacement between runs) was used to assess the spatial uncertainty of the predictions, shown here as the standard deviation of habitat suitability scores across all ten model runs. This approach does not account for all possible sources of uncertainty, but provides a useful spatial measure of how sensitive the model is to the sampling of occurrence data and the construction of the pseudoabsence dataset. Warmer colors indicate areas where model runs disagreed more prominently, indicating higher uncertainty in predictions. [file peerj-09-11972-s040.png]
